# Monocyte apoptotic bodies are vehicles for influenza A virus propagation

**DOI:** 10.1038/s42003-020-0955-8

**Published:** 2020-05-08

**Authors:** Georgia K. Atkin-Smith, Mubing Duan, Damien J. Zanker, Liyen Loh, Thi H. O. Nguyen, Marios Koutsakos, Tien Nguyen, Xiangrui Jiang, Julio Carrera, Thanh Kha Phan, Chuanxin Liu, Stephanie Paone, Sara Oveissi, Amy L. Hodge, Amy A. Baxter, Katherine Kedzierska, Jason M. Mackenzie, Mark D. Hulett, Pamuk Bilsel, Weisan Chen, Ivan K. H. Poon

**Affiliations:** 1 Department of Biochemistry and Genetics, La Trobe Institute for Molecular Science, La Trobe University, Melbourne, VIC 3086 Australia; 2 Department of Microbiology and Immunology, University of Melbourne, at the Peter Doherty Institute for Infection and Immunity, Parkville, VIC 3010 Australia; 3 FluGen, 597 Science Drive, Madison, WI 53711 USA

**Keywords:** Apoptosis, Influenza virus, Influenza virus

## Abstract

The disassembly of apoptotic cells into small membrane-bound vesicles termed apoptotic bodies (ApoBDs) is a hallmark of apoptosis; however, the functional significance of this process is not well defined. We recently discovered a new membrane protrusion (termed beaded apoptopodia) generated by apoptotic monocytes which fragments to release an abundance of ApoBDs. To investigate the function of apoptotic monocyte disassembly, we used influenza A virus (IAV) infection as a proof-of-concept model, as IAV commonly infects monocytes in physiological settings. We show that ApoBDs generated from IAV-infected monocytes contained IAV mRNA, protein and virions and consequently, could facilitate viral propagation in vitro and in vivo, and induce a robust antiviral immune response. We also identified an antipsychotic, Haloperidol, as an unexpected inhibitor of monocyte cell disassembly which could impair ApoBD-mediated viral propagation under in vitro conditions. Together, this study reveals a previously unrecognised function of apoptotic monocyte disassembly in the pathogenesis of IAV infections.

## Abstract (web summary)

Atkin-Smith et al. study apoptotic bodies formed when Influenza A (IAV)-infected monocytes undergo apoptosis. They find that apoptotic bodies contain components of IAV and can contribute to IAV propagation in vitro and in mice, and stimulate an innate and adaptive immune response. This study suggests that apoptotic bodies may take part in virus propagation and/or clearance.

## Introduction

Apoptosis is a form of programmed cell death that can occur as a result of physiological homoeostasis or in disease settings, including chronic inflammation and infection^1,2^. In comparison to other forms of cell death, such as necrosis and pyroptosis, apoptosis usually promotes an anti-inflammatory response^3^. Apoptotic cells can undergo a series of morphological changes to aid cell fragmentation, a process termed apoptotic cell disassembly^4^. This process can be divided into three morphologically distinct steps, including plasma membrane blebbing (step 1), thin apoptotic membrane protrusion formation (step 2), and finally fragmentation into a type of extracellular vesicles (EVs) known as apoptotic bodies (ApoBDs; step 3)^4,5^. Although ApoBD formation was generally thought to be a stochastic event, we recently demonstrated that monocytes undergo a highly coordinated and controlled disassembly process^6^. Apoptotic monocytes generate long membrane protrusions that exhibit a unique ‘beads-on-a-string’ morphology, termed beaded apoptopodia, which undergo a segmentation-like event to fragment into an abundance of ApoBDs. Furthermore, beaded apoptopodia were found to be positively regulated by vesicular trafficking and negatively regulated by the membrane channel pannexin 1^6^. Although the formation of ApoBDs has been speculated to aid the efficient clearance of apoptotic debris and intercellular communication, the function of ApoBDs are yet to be fully defined.

Influenza A virus, a negative-sense RNA virus of the *Orthomyxoviridae* family, can infect both immune and non-immune cell types, such as macrophages^7^, monocytes^8,9^, and epithelial cells^10^. As airway and alveolar epithelial cells predominately reside within the respiratory tract and lung parenchyma, they are key targets of IAV infection. Infected alveolar epithelial cells release a series of chemoattractants, such as CCL2, which recruit inflammatory monocytes to the site of infection^11^. Consequently, monocytes are often exposed to IAV during infection and undergo apoptosis^8,12,13^. Therefore, we utilised IAV infection as a model to further dissect the functional consequence of the disassembly of apoptotic monocytes. IAV was able to induce monocyte apoptosis and apoptotic cell disassembly in both in vitro and in vivo settings. ApoBDs generated by IAV-infected monocytes contained a series of IAV components, including IAV mRNA, proteins, and infectious virions. As a consequence, such ApoBDs could aid the propagation of IAV in vitro and in vivo, and stimulate an innate and adaptive immune response. Furthermore, we identified a commonly used antipsychotic, Haloperidol, as an inhibitor of apoptotic monocyte disassembly, which could impair IAV propagation via ApoBDs and lessen disease severity. Taken together, this proof-of-concept study reveals a previously undescribed function of monocyte-derived ApoBDs in an infectious disease setting.

## Results

### Induction of monocyte apoptosis and cell disassembly by IAV

**Fig. 1:**
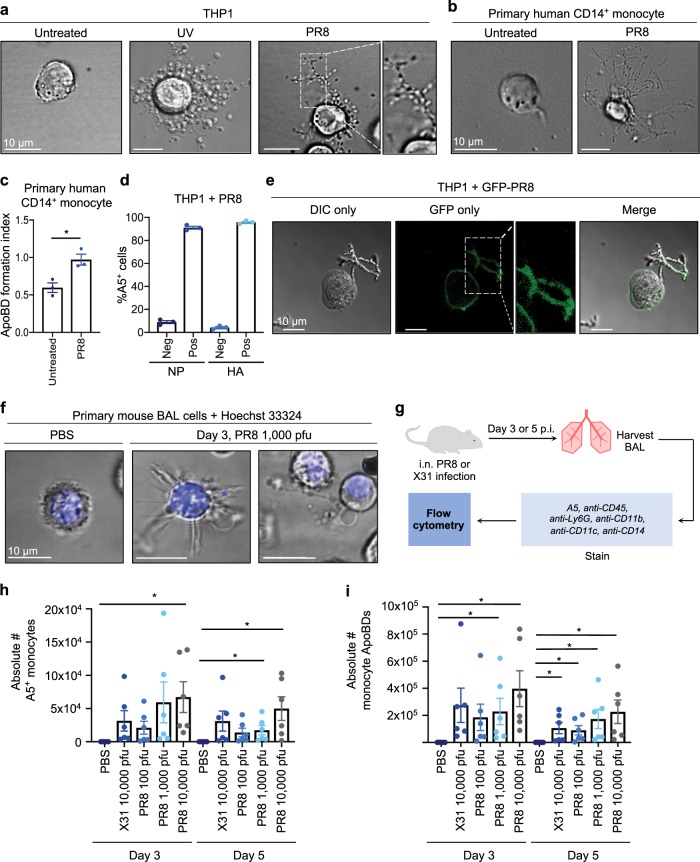
Influenza A virus induces monocyte apoptotic cell disassembly in vitro and in vivo. **a** THP1 monocytes were exposed to UV irradiation or infected with PR8, and imaged by DIC microscopy 3 and 24 h post treatment, respectively. Primary human CD14^+^ monocytes were infected with PR8 and imaged by DIC microscopy **b** or subjected to flow cytometry 24 h p.i. **c**. ApoBD formation index = number of A5^+^ ApoBDs/number of A5^+^ apoptotic cells. **d** THP1 monocytes were infected with PR8, and the percentage of NP/HA and A5-positive cells was determined by flow cytometry. **e** THP1 monocytes were infected with PR8-GFP and GFP expression was monitored by confocal microscopy, 24 h p.i. **f** B6 mice were administered PBS or 1000 pfu PR8 and BAL cells harvested 3 days p.i., stained with Hoechst and monitored by confocal microscopy (representative of *n* = 3 independent repeats). **g** Schematic diagram of a mouse IAV infection model. The total number of A5-positive mouse monocytes **h** and monocyte ApoBDs **i** within the BAL were quantified by flow cytometry under the following infection conditions; PBS, 10,000 pfu X31 and 100, 1000, or 10,000 pfu PR8 at either day 3 or day 5 p.i., error bars represent SEM (*n* = 9 mice). Unless otherwise specified, all in vitro data are representative of at least three independent experiments where error bars represent SEM of *n* = 3 biological repeats, *P < 0.05, **P < 0.01, ***P < 0.001, unpaired Student’s two-tailed *t*-test.

To first confirm that the IAV strain A/Puerto Rico/8/1934 H1N1 (PR8) could induce monocyte apoptosis in our experimental setting, we infected the human monocytic cell line THP1 with PR8 for either 6 or 24 h^14^. PR8 infection reduced THP1 monocyte viability and induced apoptosis, a process that could be limited by the presence of the pan-caspase inhibitor Q-VD-OPh (Supplementary Fig. 1a, b). Furthermore, we validated that PR8 could induce other apoptotic features, such as procaspase activation and DNA fragmentation (Supplementary Fig. 1c, d). Flow cytometry analysis demonstrated that PR8-infected THP1 monocytes generated an abundance of ApoBDs (Supplementary Fig. 1e), and beaded apoptopodia, 24 h post infection (p.i.; Fig. 1a). Similarly, PR8 infection could also induce beaded apoptopodia and ApoBD formation by primary human CD14^+^ monocytes (Fig. 1b, c). To validate that apoptotic cells were indeed infected, we confirmed the presence of the IAV proteins nucleoprotein (NP) and haemagglutinin (HA) in/on annexin A5 (A5) positive (i.e., apoptotic) THP1 cells (Fig. 1d). Additionally, we infected THP1 cells with a genetically modified PR8 virus (PR8-GFP), whereby a GFP gene is integrated into the non-structural protein (NS) gene segment and GFP will only be produced after viral replication^13^. Confocal microscopy analysis of THP1 monocytes infected with PR8-GFP demonstrated the expression of GFP 24 h p.i. (Fig. 1e), indicative of a productive infection.

We next asked whether IAV-induced apoptotic monocyte disassembly occurred in an in vivo setting. We first infected C57BL/6 (B6) mice intranasally (i.n.) with 1000 pfu PR8 and monitored the morphologies of cells within the bronchial alveolar lavage (BAL) fluid, which contains a mixture of AMΦ, inflammatory monocytes, and neutrophils. Consistent with in vitro analysis, PR8 infection induced beaded apoptopodia formation by BAL cells, 3 days p.i. (Fig. 1f, Supplementary Fig. 2). To further characterise IAV-induced monocyte cell death and ApoBD formation in vivo, we infected B6 mice with a series of PR8 titres and with the sublethal H3N2 virus, X31 (Fig. 1g). Flow cytometry analysis demonstrated that PR8 induced a titre-dependent increase in mouse monocyte cell death at both days 3 and 5 p.i., (Fig. 1h, Supplementary Fig. 3). Importantly, a significant increase of monocyte-derived ApoBDs was also observed within the BAL at both days 3 and 5 p.i. (Fig. 1i), and ApoBDs exhibited a diameter of ~2 µm (Supplementary Fig. 4), consistent with the ApoBDs generated from human monocytes^6^. It is of interest to note that both PR8 and X31 also induced cell death and the disassembly of AMΦs (Supplementary Fig. 5). Collectively, these data demonstrate that IAV can induce apoptotic monocyte disassembly thus, IAV is a suitable experimental model to examine the role of monocyte-derived ApoBDs in a physiologically relevant infection setting.

### Monocyte ApoBDs harbour IAV protein, mRNA, and virions

**Fig. 2:**
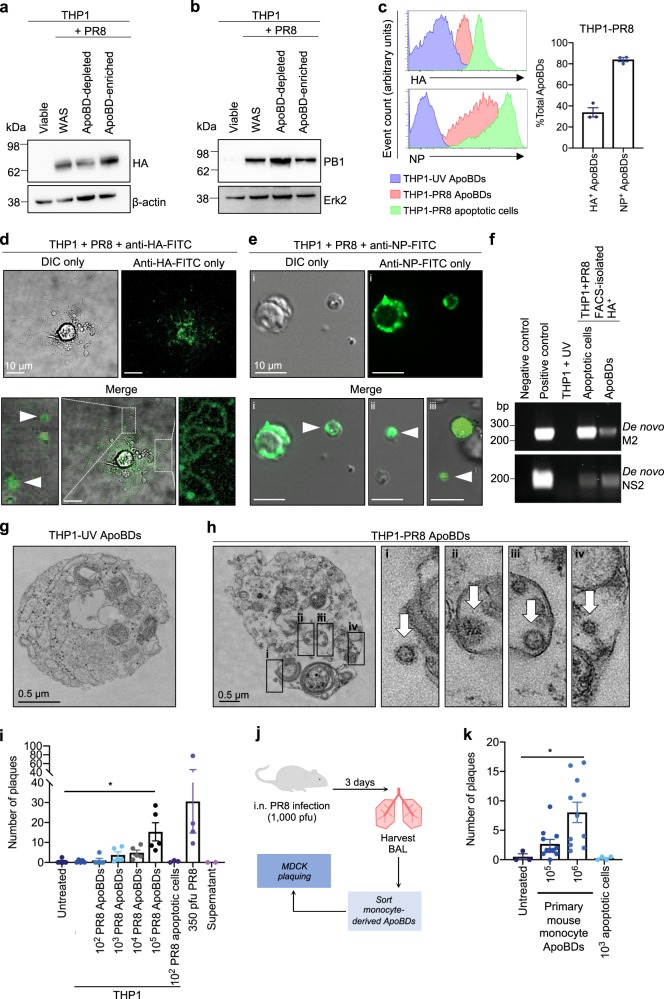
Influenza proteins, mRNA, and virions are distributed into the ApoBDs of infected monocytes. THP1 monocytes were infected with PR8 and ApoBDs were isolated 24 h p.i. via differential centrifugation for immunoblot analysis of HA **a** and PB1 **b** expression (representative of *n* = 2 independent experiments; WAS = Whole Apoptotic Sample). **c** THP1 monocytes were infected with PR8 (24 h p.i.) or subjected to UV irradiation (4 h), and ApoBDs and apoptotic cells were analysed for the presence of HA and NP by flow cytometry. **d** Live cell confocal microscopy was performed to monitor the distribution of HA on PR8-infected THP1 monocytes (24 h p.i.). **e** NP staining was monitored by confocal microscopy on PR8-infected THP1 monocytes (24 h p.i.). **f** PCR analysis of the 3ʹ spliced junction of IAV M2 and NS2 was performed on FACS-isolated HA^+^ apoptotic cells and ApoBDs. Transmission electron microscopy was performed on ApoBDs derived from UV-irradiated (4 h post UV) **g** or PR8-infected (24 h p.i.) **h** THP1 monocytes. **i** FACS-isolated ApoBDs from UV-treated or PR8-infected THP1 monocytes were subjected to viral plaquing (dots represent average of *n* = 2 biological repeats). **j** Schematic diagram of methodology used for plaque assay on primary monocyte ApoBDs. **k** Primary mouse monocyte ApoBDs were isolated via FACS from PR8-infected mice (1000 pfu, day 3 p.i.) and subjected to viral plaquing (dots represent average of *n* = 2 biological repeats). Unless otherwise specified, data shown is representative three independent experiments, *P < 0.05, unpaired Student’s two-tailed *t*-test.

Next, we examined the distribution of IAV biomolecules in/on the ApoBDs of IAV-infected monocytes. ApoBDs from PR8-infected THP1 monocytes were isolated^15^ (Supplementary Fig. 6), and the expression of the viral proteins HA and PB1 were confirmed in the ApoBD-enriched fraction by immunoblotting (Fig. 2a, b). Furthermore, the expression of IAV HA and NP on/in the ApoBDs, and apoptopodia of PR8-infected THP1 monocytes was also confirmed by flow cytometry and confocal microscopy (Fig. 2c–e). To determine whether ApoBDs also contained IAV mRNA, total mRNA was extracted from FACS-isolated HA^+^ ApoBDs from PR8-infected THP1 monocytes for PCR analysis. A prominent PCR product was observed for the IAV matrix gene segment (M) and NS, when a forward primer complimenting the 3ʹ spliced junction of either gene was used in the PCR reaction (Fig. 2f). As NS2 and M2 mRNA are generated specifically through mRNA splicing following viral replication and absent within the IAV virion itself, detection of the 3ʹ spliced junction of NS2 and M2 specifically indicates a productive infection, and the subsequent segregation of IAV mRNA into ApoBDs, rather than RNA carryover from fully assembled virions.

To address whether ApoBDs generated from PR8-infected THP1 monocytes could harbour virions, we performed transmission electron microscopy analysis on ApoBDs derived from UV-irradiated and PR8-infected cells. In contrast to the UV-THP1 ApoBDs (Fig. 2g), IAV virions could be observed both externally, associated with the ApoBDs (Fig. 2h(i)), and also internally, within the ApoBDs (Fig. 2h(ii–iv)) of PR8-infected THP1 monocytes. Thus, to determine whether the ApoBDs derived from PR8-infected cells were infectious, we next performed Madin-Darby canine kidney (MDCK)-viral plaquing^16^. The isolation of ApoBDs was first validated to confirm the efficiency of our previously described FACS-based ApoBD isolation approach (Supplementary Fig. 7a, b)^15,17^. ApoBDs generated from PR8-infected THP1 monocytes induced a concentration-dependent increase in plaque formation, in contrast to the ApoBDs generated from UV-irradiated THP1 monocytes and the untreated control (Fig. 2i). Importantly, the supernatant control (i.e., supernatant collected from FACS-isolated ApoBDs without ApoBDs) did not induce plaque formation, indicating that free-floating virions are unlikely to be carried over from the initial infection during the ApoBD isolation process (Fig. 2i). Next, we performed MDCK-viral plaquing on FACS-isolated mouse monocyte-derived ApoBDs (isolated from the BAL of B6 mice infected with 1000 pfu PR8, day 3 p.i.; Fig. 2j). Mouse-derived ApoBDs were isolated to ~90% purity, whereby 98% of all isolated ApoBDs were of monocyte origin (Supplementary Fig. 8). Consistent with PR8-THP1 ApoBDs, monocyte-derived ApoBDs from PR8-infected mice could also induce plaque formation (Fig. 2k). Together, these results demonstrate that viral mRNA, proteins, and infectious virions are distributed in/on the ApoBDs of IAV-infected apoptotic monocytes.

### ApoBDs can facilitate viral propagation in vitro

**Fig. 3:**
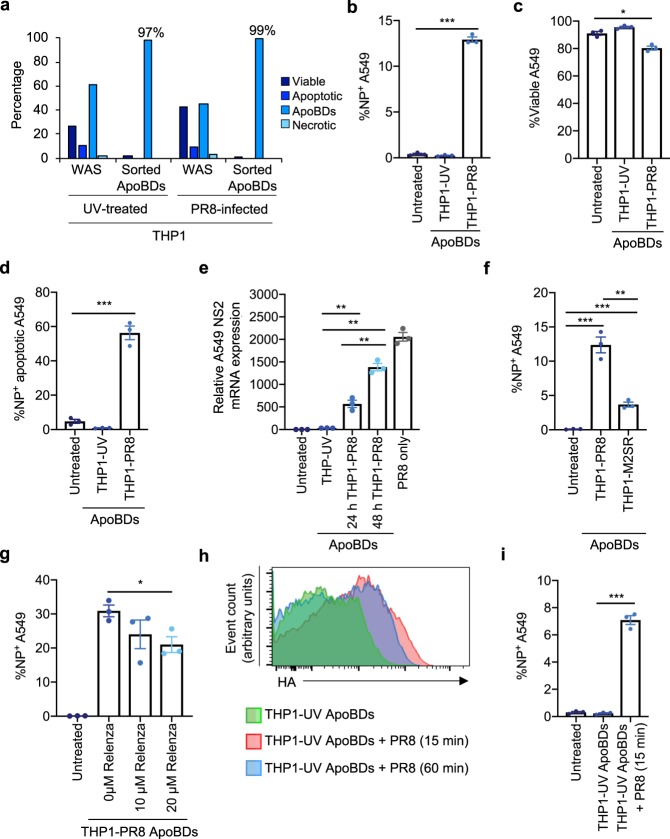
ApoBDs from IAV-infected THP1 monocytes can aid viral propagation in vitro. **a** Purity of FACS-based ApoBD isolation approach depicting the levels of viable, apoptotic, and necrotic cells, and ApoBDs before and after sorting. ApoBDs (1 × 10^5^) from PR8-infected cells were incubated with A549 epithelial cells for 48 h, and flow cytometry was performed to determine: **b** the percentage of NP^+^ A549 cells, **c** the percentage of viable A549 cells, and **d** the percentage of NP^+^ apoptotic A549 cells. **e** RT-qPCR analysis was performed on A549 cells subjected to 1 × 10^5^ ApoBDs for 24 or 48 h (UV-irradiated or PR8-infected THP1 ApoBDs). Relative expression was normalised to the house keeping gene, UBC. **f** A549 epithelial cells were incubated with 1 × 10^5^ ApoBDs isolated from PR8- or M2SR-infected THP1 monocytes for 48 h, and the percent NP^+^ A549 cells was determined by flow cytometry. **g** A549 epithelial cells were treated with 1 × 10^5^ ApoBDs for 48 h in the presence of 10 or 20 µM Relenza and NP expression was determined by flow cytometry. **h** Apoptotic (UV-irradiated) THP1 monocytes were incubated with PR8 for 15 or 60 min, and ApoBDs isolated by differential centrifugation were analysed for HA staining. **i** A549 cells were co-incubated with PR8-exposed, FACS-isolated ApoBDs from THP1-UV, and flow cytometry was performed to determine A549 NP expression. Unless otherwise specified, error bars represent SEM of *n* = 3 biological repeats, presented data are representative of at least three independent experiments, *P < 0.05, **P < 0.01, ***P < 0.001, unpaired Student’s two-tailed *t*-test.

As IAV infection can induce the formation of monocyte ApoBDs that harbour IAV components, we next asked whether ApoBDs could mediate viral propagation. ApoBDs were isolated from UV-irradiated or PR8-infected THP1 monocytes (Fig. 3a). ApoBDs were then co-incubated with target cells (human lung epithelial A549 cells) for 48 h, and target cells were assessed for NP expression and cell viability (indicative of viral infection) by flow cytometry. A549 target cells co-incubated with THP1-PR8 ApoBDs exhibited a significant increase in NP expression, compared to the untreated A549 cell only control or co-incubation with THP1-UV ApoBDs (Fig. 3b). Furthermore, THP1-PR8 ApoBD co-incubation also reduced A549 target cell viability and resultant apoptotic A549 target cells exhibited NP expression, indicating that the dying cells were infected with IAV (Fig. 3c, d). To determine whether THP1-PR8 ApoBDs were able to induce a productive IAV infection in A549 target cells, we confirmed the presence of the 3ʹ spliced junction of NS2 mRNA in A549 target cells by RT-qPCR, indicating that the ApoBDs from PR8-infected THP1 monocytes could induce a productive IAV infection (Fig. 3e).

Next, we aimed to investigate the mechanism of ApoBD-mediated viral propagation. First, we assessed the contribution of viral budding in this process by infecting THP1 monocytes with M2SR, an M2-deficient PR8 virus. Cells infected with M2SR express all IAV proteins except for the M2 ion channel, a protein important for virion assembly, and therefore cannot produce infectious virions following the first round of infection^18^. M2SR-infected THP1 monocytes were able to undergo apoptosis and generated a comparable level of ApoBDs as PR8-infected cells (Supplementary Fig. 9a). ApoBDs generated from M2SR-infected THP1 monocytes also exhibited a comparable level of HA and NP expression to the THP1-PR8 ApoBDs (Supplementary Fig. 9b), indicating that the induction of monocyte apoptosis, apoptotic cell disassembly, and the distribution of certain viral proteins into ApoBDs is not affected by the absence of a functional M2 protein. However, when performing co-incubation assays, the ApoBDs from M2SR-infected THP1 monocytes were significantly impaired in their ability to induce target cell infection, in comparison to those generated from PR8-infected THP1 cells (Fig. 3f). During IAV infection, NA cleaves host cell surface sialic acid residues to release progeny virions from the plasma membrane. Therefore, we asked whether the pharmaceutical compound Relenza, which antagonises the activity of NA^19^, could interfere with viral dissemination from ApoBDs. Treatment of THP1-PR8 ApoBDs co-incubated with A549 target cells with Relenza resulted in a concentration-dependent decrease in A549 cell NP expression (Fig. 3g). Together, these results indicate that the mechanism of ApoBD-mediated viral propagation is primarily dependent on the capacity to generate viral progeny.

Lastly, we asked whether newly generated IAV virions could bind directly to the surface of ApoBDs, and whether ApoBDs could then act as a cell surface vector and transfer the infectious virions to neighbouring cells. Apoptotic THP1 monocytes (UV irradiated) were incubated with PR8 for a short duration to allow infectious virions to bind to newly formed ApoBDs (without necessarily inducing a productive infection). Importantly, HA^+^ ApoBDs (indicative of virion binding) was first validated by flow cytometry, with no marked differences observed between samples incubated with PR8 for 60 or 15 min (Fig. 3h). This suggests that virions were likely to be retained on the ApoBD surface as virion uptake would result in a decrease in HA^+^ staining. Next, we isolated ApoBDs from UV-irradiated apoptotic samples that had been incubated with PR8 and co-incubated them with A549 target cells. PR8-treated ApoBDs were able to induce a significant increase in A549 target cell NP expression, compared to untreated ApoBDs (Fig. 3i). Together, these data demonstrate that the ApoBDs from IAV-infected THP1 monocytes are able to induce a productive infection in recipient cells, primarily through the trafficking of infectious virions.

### Monocyte-derived ApoBDs facilitate viral propagation in vivo

**Fig. 4:**
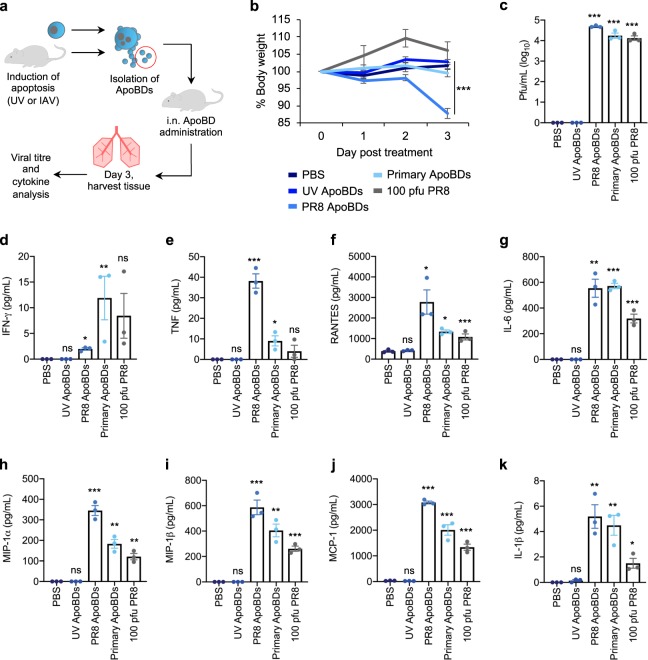
Monocyte-derived ApoBDs can aid viral propagation in vivo. **a** Schematic diagram of ApoBD isolation and in vivo administration. **b** Mice were treated with PBS, ApoBDs (UV-THP1, PR8-THP1, or primary mouse monocyte ApoBDs), or 100 pfu PR8, and body weight was monitored over 3 days post treatment. Lung tissue was collected on day 3 post treatment and lung viral titres were determined by MDCK plaquing **c**, and inflammatory cytokine production was determined by cytometric bead array **d**–**k**. Error bars represent SEM (*n* = 3 mice), ns = *P* > 0.05, *P < 0.05, **P < 0.01, ***P < 0.001, unpaired Student’s two-tailed *t*-test. Statistical significance of UV-ApoBD, PR8 ApoBD, Primary-ApoBD, and PR8 treatment was determined by comparison to the PBS only sample.

Next, we aimed to determine whether ApoBDs could also facilitate the propagation of IAV in vivo. As 100 pfu PR8 could induce monocyte apoptotic cell disassembly in vivo and generate >1 × 10^5^ ApoBDs by day 3 p.i (Fig. 1i), we chose to administer 1 × 10^5^ THP1-PR8 ApoBDs into mice and compare their responses to 100 pfu PR8-infected control B6 mice, as a physiologically relevant model of high-pathogenicity IAV infection (Fig. 4a). We also treated mice with 4 × 10^4^ primary monocyte-derived ApoBDs, denoted as Primary ApoBDs, isolated from the BAL of PR8-infected mice (1000 pfu, day 3). The purity of our ApoBD isolation approach was first validated by flow cytometry (Supplementary Fig. 10a–c) and mouse body weight was monitored daily following administration of ApoBDs as a surrogate measure of IAV-induced disease severity. By day 3 post treatment, mice treated with THP1-PR8 ApoBDs exhibited a significant decrease in body weight, indicating the onset of disease (Fig. 4b). Lung tissue was harvested on day 3 for viral titre analysis, which demonstrated that treatment with THP1-PR8 ApoBDs or ApoBDs from primary monocytes elicited significant viral titres within the lung, comparable to that of infection with 100 pfu PR8 (Fig. 4c). As IAV infection and propagation is associated with a robust inflammatory response, we next quantified the secretion of inflammatory cytokines within the lung. In comparison to treatment with the ApoBDs derived from UV-irradiated THP1 monocytes, THP1-PR8 ApoBDs and primary ApoBDs elicited a significant inflammatory response, as shown by increased levels in IFN-γ, TNF, RANTES, IL-6, MIP-1α, MIP-1β, MCP-1, and IL-1β, similar to the 100 pfu PR8 alone condition (Fig. 4d–k). Together, these data demonstrate that the ApoBDs of IAV-infected monocytes can propagate IAV and induce a robust infection in vivo.

### ApoBDs induce a robust adaptive anti-IAV immune response

To further characterise the ability of ApoBDs to propagate IAV infection in vivo, we aimed to examine whether THP1-PR8 ApoBDs could elicit an IAV-specific adaptive immune response. We administered ApoBDs isolated from UV-treated, PR8- or M2SR-infected THP1 monocytes i.n. into B6 mice and harvested tissue at day 8, when the presence of an adaptive immune response is prominent. As above, the purity of our ApoBD isolation approach was validated and mouse body weight was measured daily post ApoBD treatment, whereby only mice treated with either THP1-PR8 ApoBDs or 100 pfu PR8 exhibited a significant loss in body weight by day 8 p.i., indicative of a severe infection (Supplementary Fig. 11a, b). We first validated immune cell recruitment where administration of THP1-PR8 ApoBDs or 100 pfu PR8 both induced the preferential recruitment of CD8^+^ T cells, as well as the recruitment of monocytes and neutrophils into the lung parenchyma (Supplementary Fig. 11c–e). In contrast, mice treated with THP1-UV ApoBDs or THP1-M2SR ApoBDs lacked immune cell recruitment.

**Fig. 5:**
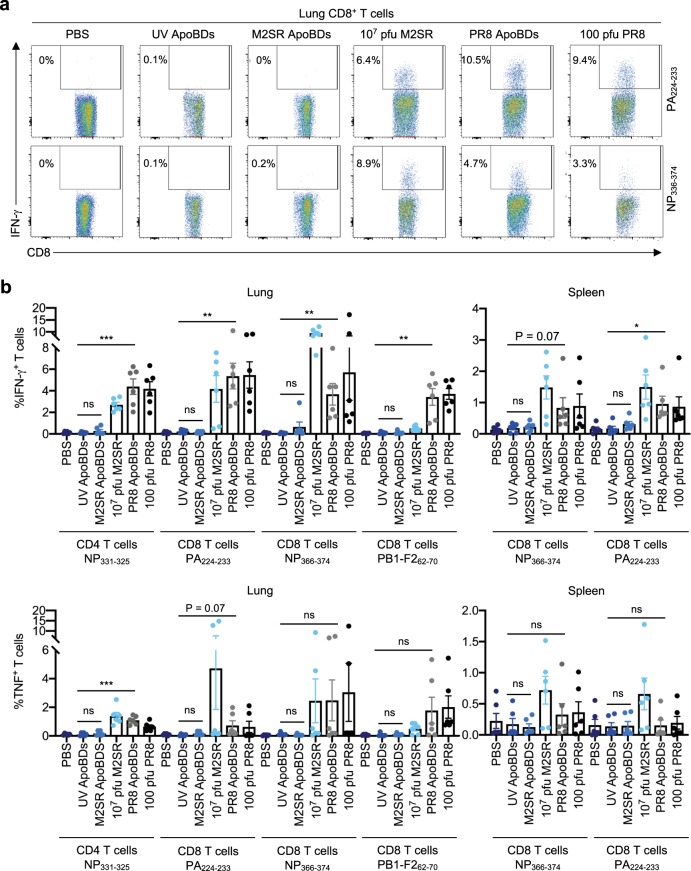
ApoBDs from IAV-infected THP1 monocytes stimulate a robust IAV-specific T cell response. **a**, **b** Mice were administered with PBS, 10^7^ pfu M2SR, 100 pfu PR8, UV-THP1 ApoBDs, M2SR-THP1 ApoBDs, or PR8-THP1 ApoBDs. Eight days post administration, ICS was performed on lung- and splenic-derived CD4^+^ and CD8^+^ T cells after incubation with the following IAV peptides: NP_311–325_, NP_366–374_, PA_224–233_, and PB1F2_62–70_. IFN-γ and TNF expression was quantified by flow cytometry. Error bars represent SEM (*n* = 5–6 mice).

Next, we performed intracellular cytokine staining (ICS), and quantified IFN-γ and TNF-producing CD8^+^ and CD4^+^ T cells derived from the lung and spleen after stimulation with an array of IAV peptides (Fig. 5a, b). The presentation of viral-derived peptides to T cells in vivo induces T cell priming, and expansion to generate IAV-specific CD8^+^ and CD4^+^ T cells. Therefore, ex vivo re-stimulation of T cells with IAV peptides will lead to IFN-γ/TNF production by IAV-specific T cells. As expected, B6 mice treated with PBS or THP1-UV ApoBDs did not harbour detectable IAV-specific CD8^+^ and CD4^+^ T cells (Fig. 5a, b). In contrast, lung-derived CD8^+^ and CD4^+^ T cells from mice treated with THP1-PR8 ApoBDs elicited significant IFN-γ and TNF expression after stimulation with either immunodominant or sub-dominant CD8^+^ T cell peptides (NP_366–374_, PA_224–233_, and PB1-F2_62–70_) or an immunodominant CD4^+^ T cell peptide (NP_311–325_; Fig. 5a, b). Similar trends were also observed in splenic T cells (Fig. 5b), altogether demonstrating that the administration of THP1-PR8 ApoBDs could promote IAV antigen presentation, and the generation of IAV-specific CD8^+^ and CD4^+^ T cells in vivo. Mice treated with 10^7^ pfu M2SR developed robust CD8^+^ T cell responses as previously reported^18^. However, IAV-specific CD8^+^ or CD4^+^ T cells were rarely generated from THP1-M2SR ApoBD-treated mice (Fig. 5a, b). As a significantly higher dose of M2SR IAV is required to induce CD8^+^ T cell activation, it is possible that the administration of >1 × 10^5^ THP1-M2SR ApoBDs may elicit a CD8^+^ T cell response. Nevertheless, the striking difference in responses between THP1-M2SR and THP1-PR8 ApoBD treatment highlights that the generation of viral progeny by ApoBDs, rather than their engulfment, facilitates the generation of anti-IAV immunity. Altogether, our data indicate that the ApoBDs from IAV-infected monocytes can aid viral propagation in vivo and elicit innate and adaptive antiviral immune responses.

### Inhibiting apoptotic cell disassembly limits IAV propagation

**Fig. 6:**
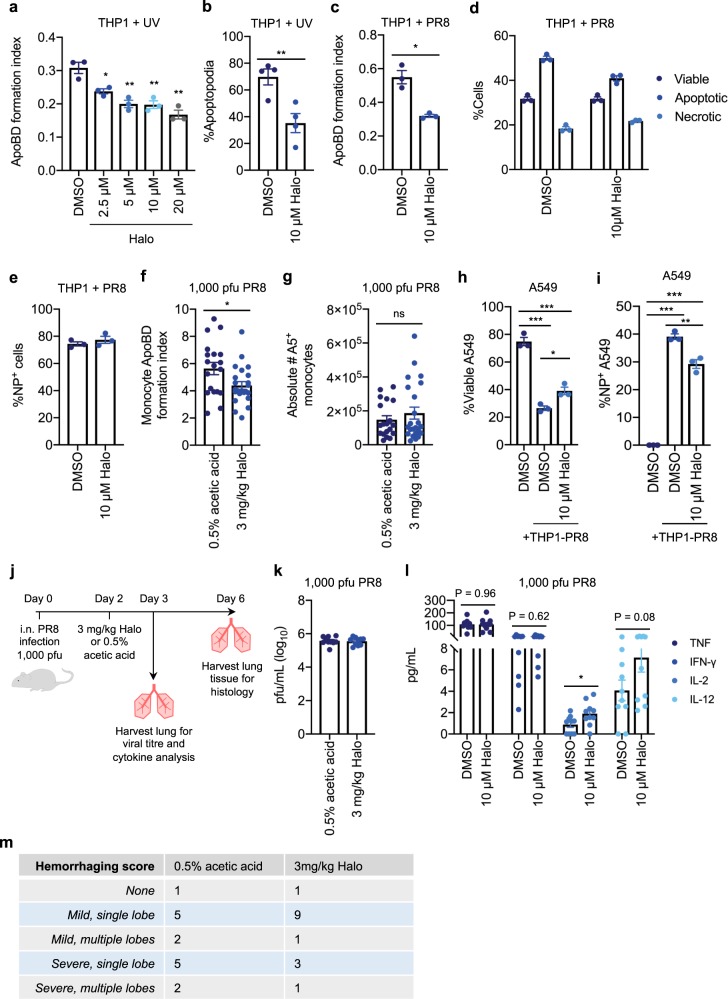
Halo is a potent inhibitor of apoptotic cell disassembly and can limit viral propagation via ApoBDs. **a** UV-irradiated THP1 monocytes were treated with 2.5–20 µM Halo for 4 h and the ApoBD formation index was determined by flow cytometry. **b** UV-irradiated THP1 monocytes were monitored by time-lapse DIC microscopy for 4 h in the presence of DMSO or 10 µM Halo and the percentage of cells forming apoptopodia was quantified (*n* = 4 independent experiments). THP1 monocytes were infected with PR8 for 24 h in the presence of DMSO or 10 µM Halo, and flow cytometry was performed to quantify the: **c** ApoBD formation index, **d** levels of viable, apoptotic, and necrotic cells, and **e** percentage of NP^+^ cells. PR8-infected (1000 pfu) mice were treated with either 0.5% acetic acid (vehicle control) or 3 mg/kg Halo on day 2 p.i. for 24 h, and flow cytometry was performed to quantify the monocyte ApoBD formation index (number of A5^+^ ApoBDs/number of A5^+^ cells) **f**, and the total number of A5^+^ monocytes within the BAL **g** (control *n* = 18 and Halo treatment *n* = 24 mice). PR8-infected THP1 monocytes were incubated with viable A549 epithelial cells for 48 h in the presence of DMSO or 10 µM Halo, and flow cytometry was performed to quantify the percentage of viable A549 cells **h** and percentage of NP^+^ A549 cells **i**. **j** Schematic of in vivo infection mouse model. Mice were infected with 1000 pfu PR8 and treated with 0.5% acetic acid or 3 mg/kg Halo on day 2 p.i., and lungs were harvested on day 3 p.i for viral titre (**k**, *n* = 10 mice) and cytokine analysis (**l**, *n* = 10 mice), or on day 6 p.i. for histological analysis (**m**, *n* = 13-14 mice). Histology slides were scored blind to determine the level of tissue haemorrhaging. Unless otherwise specified, error bars represent SEM of *n* = 3 biological repeats, presented data are representative of at least three independent experiments, *P < 0.05, **P < 0.01, ***P < 0.001, unpaired Student’s two-tailed *t*-test.

Finally, to validate our proof-of-concept model where ApoBDs can traffic infectious virions and propagate infection, we asked whether manipulation of apoptotic cell disassembly could alter viral propagation. We aimed to develop a pharmacological approach to assess whether specifically inhibiting ApoBD formation could impair IAV propagation to surrounding cells. Previously, we performed a LOPAC^1280^ drug screen on apoptotic cells and identified the antidepressant Sertraline (Zoloft), as a potent inhibitor of T cell and monocyte apoptotic cell disassembly^6,20^. Although sertraline could effectively inhibit ApoBD formation by PR8-infected THP1 monocytes, it was not suitable for this study as it also altered the levels of apoptosis and necrosis following PR8 infection (Supplementary Fig. 12a, b). We have also investigated two other commonly used antidepressants Fluvoxamine and Paroxetine, which although identified as inhibitors of THP1 monocyte ApoBD formation, these drugs significantly impaired the basal level of PR8-induced apoptosis (Supplementary Fig. 12c–h). Therefore, we next chose to investigate an antipsychotic known as Haloperidol (Halo or Haldol) initially identified in our LOPAC^1280^ drug screen. Halo was able to impair both ApoBD and apoptopodia formation by UV-irradiated THP1 monocytes (Fig. 6a, b). Additionally, Halo could also inhibit ApoBD formation by PR8-infected THP1 monocytes, without altering the levels of apoptosis and necrosis, or the level of infection as measured by NP staining (Fig. 6c–e). Therefore, we asked whether Halo could also impair monocyte apoptotic cell disassembly in vivo by treating PR8-infected B6 mice with Halo (3 mg/kg) on day 2 p.i. The BAL was collected on day 3 p.i. for flow cytometry analysis and demonstrated that Halo could reduce the formation of monocyte ApoBDs without reducing the proportion of A5^+^ monocytes (Fig. 6f, g). Together, this data demonstrates that Halo is an effective inhibitor of monocyte apoptotic cell disassembly, which does not alter other key parameters during IAV infection.

Next, we used Halo in our in vitro model to determine whether impairing apoptotic cell disassembly could reduce viral propagation, by co-incubating PR8-infected THP1 monocytes with A549 epithelial target cells for 48 h, with or without Halo treatment. Notably, 10 µM Halo partially restored the total proportion of viable A549 target cells and reduced the levels of NP-expressing A549 cells (Fig. 6h, i). Therefore, inhibition of apoptotic cell disassembly can limit the propagation of IAV in vitro. Finally, to examine the physiological consequence of monocyte ApoBD formation during IAV infection in vivo, we treated PR8-infected B6 mice (1000 pfu) with 3 mg/kg of Halo or a vehicle control (0.5% acetic acid) on day 2 p.i, and monitored viral titres, cytokine levels, and lung haemorraghing as a direct measure of disease severity (Fig. 6j). Treating IAV-infected mice with Halo had no significant effect on total lung viral titres (Fig. 6k). However, in comparison to pro-inflammatory cytokines, such as TNF and IFN-γ that remained unchanged, Halo treatment resulted in an increase in IL-2 and IL-12 (Fig. 6l), key cytokines required to boost T cell-mediated antiviral responses^21–24^. Moreover, in comparison to the vehicle control group that often had severe tissue haemorrhaging following IAV infection, the majority of IAV-infected Halo-treated mice exhibited only mild levels of haemorrhaging (Fig. 6m, tissue sections scored blind as possessing either no, mild (single or multiple lobe), or severe (single or multiple lobe) tissue haemorrhaging (Supplementary Fig. 13)). Thus, although Halo treatment could only partially reduce ApoBD formation in vivo, these data suggest that impairment of monocyte ApoBD formation may provide a protective effect during IAV infection. Taken together, our proof-of-concept study demonstrates that apoptotic monocyte disassembly can generate ApoBDs capable of aiding viral propagation, a process that can be impaired by pharmacologically targeting ApoBD formation.

## Discussion

Although the formation of ApoBDs was previously thought to be a stochastic event, recent findings demonstrate that ApoBDs are generated through a stepwise and regulated process, often involving the generation of apoptopodia^4^. Despite these mechanistic advances^6,20,25,26^, the functional consequence of generating ApoBDs, especially through monocyte beaded apoptopodia formation, has remained unclear. By utilising IAV infection as a model system, here we demonstrate that the disassembly of apoptotic monocytes can facilitate viral propagation.

ApoBDs have been shown to mediate intercellular communication in other experimental models through trafficking biomolecules, such as DNA^27^, microRNAs^28^, and host cell proteins^29^ and HIV proteins^30^. In line with this, here we show that IAV-infected THP1 monocytes generate beaded apoptopodia and ApoBDs that harbour IAV mRNAs and proteins. Therefore, the formation of ApoBDs may have important implications in antiviral immunity. For example, the trafficking of IAV proteins may facilitate viral detection by immune cells to elicit an adaptive immune response and, the uptake of viral mRNA may result in the expression of IAV proteins. Interestingly, although the ApoBDs from IAV-infected THP1 monocytes were able to elicit an IAV-specific T cell response in vivo, this response was markedly abolished when ApoBDs were derived from M2SR-infected cells, which contain equivalent levels of viral proteins (NP and HA), but are unable to assemble and disseminate viral progeny. These results provide an interesting contrast to previous studies that suggested a role of apoptotic cells in mediating T cell responses during both influenza infection^31,32^ and tuberculosis^33^ by transferring pathogen-derived proteins. Although it is clear that ApoBDs can harbour IAV proteins, a greater number of ApoBDs than utilised in this study may be required to elicit a robust immune response. In support of this, it is important to highlight that a significantly high pfu of M2SR virus is required to elicit a robust adaptive immune response in vivo.

Our results demonstrate that the ApoBDs from IAV-infected monocytes contained/carried IAV, and were able to propagate infection in vitro and in vivo. ApoBD-mediated viral propagation was primarily dependent on the generation and/or trafficking of IAV progeny by the ApoBDs, as pharmaceutical (Relenza treatment) or genetic (M2SR-mutant IAV) inhibition of viral dissemination was sufficient to impair IAV propagation via ApoBDs. The generation of ApoBDs may provide more surfaces/vehicles for previously generated virions to re-attach to and ApoBDs may traffic multiple virions simultaneously (Fig. 2h) thus, providing an effective method of trafficking. Furthermore, in contrast to ‘free’ virus, an ApoBD–IAV complex is likely to aid viral propagation through directly interacting with bystander cells via virion HA molecules and/or through ‘eat-me’ signals, such as PtdSer present on the ApoBD surface. Therefore, whether ApoBD-mediated viral propagation is dependent on, or even limited by, efferocytosis would be of interest to determine. Notably, the engulfment of apoptotic debris during IAV infection has been suggested to be important in disease resolution^34^ and cross-presentation^31,35^. Although apoptotic cells may also possess similar qualities to ApoBDs, the disassembly of apoptotic monocytes generates an abundance of ApoBDs, and thus may create up to ten times more infectious vehicles that have the potential to spread and propagate IAV infection. Notably, whether other EVs, in particular small EVs (e.g., exosomes) could also possess similar functional properties during IAV infection would be interesting to determine in future studies. Moreover, given that a wide variety of cells, such as monocytes, macrophages, and epithelial cells are susceptible to IAV-induced apoptosis^1^, whether other cell types in addition to monocytes, or potentially different subsets of monocytes (e.g., inflammatory vs regulatory) can disassemble into ApoBDs that aid viral propagation would also be of significant biological interest. Notably, here we did observe the ability of AMΦs to disassemble into ApoBDs during IAV infection (Supplementary Fig. 5b). Overall, the pro-viral role of apoptosis is now becoming increasingly clear where the induction of apoptosis by IAV can facilitate viral replication and/or dissemination, resulting in enhanced viral titres^1,36–38^. Additionally, impairment of apoptosis (such as through the loss of the pro-apoptotic IAV protein PB1-F2) has been shown to aid disease resolution^1,39^.

Finally, here we characterised the commonly prescribed antipsychotic Halo as a previously undescribed inhibitor of apoptotic cell disassembly. Although Halo was previously shown to have cytotoxic effects at high concentrations^40^, our results demonstrate that Halo could significantly impair the formation of beaded apoptopodia and fragmentation into ApoBDs by UV-irradiated or IAV-infected THP1 monocytes. Moreover, to our knowledge, treatment of IAV-infected mice with Halo revealed the first pharmaceutical compound that can limit the formation of ApoBDs in vivo and consequentially, lessen IAV-induced disease pathology. Clinically, Halo is prescribed for psychotic conditions, such as schizophrenia by acting as a dopamine receptor (D_2_R) antagonist^41–43^. Although in in vitro assays Halo can target D_2_Rs at low concentrations (2 nM–1 µM)^44,45^, monocytes exhibit low D_2_R expression^46^; therefore, whether Halo can impair monocyte apoptotic cell disassembly through targeting D_2_Rs is yet to be determined. Thus, further mechanistic analysis investigating how Halo can alter ApoBD formation would be of interest and may shed important light on the elusive molecular control underpinning apoptotic cell disassembly. Together, although Halo is commonly associated with a series of side effects in patients^43,47,48^ and may not be preferential for antiviral therapeutics, the use of Halo in this study further supports our proof-of-concept model, whereby the disassembly of apoptotic monocytes can aid the propagation of IAV in vitro. Notably, the identification of additional compounds that can inhibit ApoBD formation with greater potency may provide exciting therapeutic potential and worth pursuing in future studies. Altogether, although important for the efficient removal of infected cells, the induction of apoptosis by IAV is likely to represent a ‘double-edge sword’ phenomenon and contribute to both viral pathogenesis and clearance.

## Methods

### Reagents

Sertraline, Haloperidol, Paroxetine, Fluvoxamine, and EDTA were purchased from Sigma-Aldrich (St Louis, USA). The following regents were purchased from BD Biosciences (Auckland, New Zealand): A5-FITC, -PE, -APC, A5 binding buffer, mouse CD4-APC Cy7, -PE Cy7, anti-mouse CD3-APC, anti-mouse CD45.2 PerCP Cy5.5, anti-mouse Ly6G-PE, TNFα-FITC, RPMI 1640 medium, penicillin, and streptomycin. Anti-mouse CD8a-e450, anti-mouse CD11b-PE Cy7, TO-PRO-3 iodide, PO-PRO-1, hoechst 33342, NP-FITC, Live Dead-APC, -Yellow, and foetal calf serum (FCS) were purchased from Thermofisher (Scoresby, Australia). Anti-mouse CD14-PerCP Cy5.5, anti-mouse CD45.2-FITC, anti-mouse CD8-PE, and anti-mouse CD11c-APC Cy7 were purchased form Biolegend (San Diego, USA). HA-FITC was purchased from Santa Cruz Biotehnology (Texas, USA), and NP-FITC was purchased from Invitrogen (Carlsbad, USA). Anti-IFN-γ was purchased from Tonbo Biosciences (Ferris Square, USA), and MycoZap reagent was purchased from Lonza (Basel, Switzerland). Filcol Paque Premium was purchased from GE Healthcare Life Science (Chicago, USA).

### Mice

Female C57BL/6 mice were purchased from the Walter Eliza Hall Institute of Medical Research (Melbourne, Australia). All mice were housed in specific pathogen-free (SPF) isolators and infected with IAV at 6–8 weeks of age under ethics approval of AEC15-84. All experiments were approved by the La Trobe University Animal Ethics Committee in accordance with the National Health and Medical Research Council Australia code of practice for the care and use of animals for scientific purposes.

### Mammalian cell culture

THP1 monocytes and A549 epithelial cells were cultured in complete (10%) RPMI consisting of RPMI 1640 medium, 50 IU/mL penicillin and 50 µg/mL streptomycin mixture, and 10% (vol/vol) FSC. THP1 monocytes were also cultured in the presence of 0.2% (vol/vol) MycoZap reagent. Primary human monocytes were isolated from whole blood from healthy donors (Australian Red Cross Blood Service, agreement number: 14-11 VIC-03, La Trobe University Human Ethics: FHEC09/R16) through Filcol isolation and CD14 MicroBeads, in accordance to the manufactures instructions (Miltenyi Biotec, Bergisch Gladbach, Germany). All cell lines were incubated at 37 °C in 5% CO_2_.

### UV-induced apoptosis

To induce apoptosis via UV irradiation, cells were exposed to irradiation using the Stratagene UV Stratalinker 1800 (Aglient Technologies, Santa Clara, USA) at 150 mJ/cm^2^ and incubated for 2–4 h or as specified.

### IAV infections

WT-PR8 (A/Puerto Rico/8/1934 H1N1), PR8-GFP (a kind gift from Dr Adolfo García-Sastre, Mount Sinai School of Medicine, New York^13^), and X31 were propagated in SPF embryonated hen eggs; PR8-M2SR, in M2CK cells as previously described^18^. Viral titres were determined through viral plaquing as outlined below. THP1 monocytes were infected at a multiplicity of infection of 10 in acidified medium for 1 h and incubated for 24 h unless otherwise specified. Infections of mice were performed by administering specified pfu (100–10,000) of PR8 or X31, or 10^7^ pfu M2SR in 30 µL PBS, i.n. under methoxyflurane-induced anaesthesia. As M2SR virions are restricted to one round of infection, a significantly higher pfu of M2SR (compared to PR8) is necessary to ensure robust innate and adaptive immune cell readouts.

### Flow cytometry and FACS

THP1 and primary monocyte flow cytometry and FACS assays were performed as per Jiang et al. and Atkin-Smith et al., using A5 and TO-PRO-3-based analysis^15,49^. HA staining was performed using 1:250 HA-FITC, in A5 binding buffer for 20 min on ice. When fixation was required, analysis was performed using Live Dead-Yellow/APC rather than TO-PRO-3. Fixation was performed using 1% paraformaldehyde (PFA). For NP-FITC staining, samples were first permeabilised in 0.1% saponin and fixed, prior to staining at 1:50 in 0.1% saponin for 25 min at RT. For flow cytometric analysis of primary BAL samples, mice were euthanized at days 3 or 5 p.i. with a subcutaneous injection of lethabarb (320 mg/mL). To collect the BAL, the trachea was cannulated and 4 × 0.4 mL aliquots of lavage buffer (10% FCS, 2 mM EDTA, and 1× PBS) was delivered to and retrieved from the lungs. To analyse cell death and disassembly in the lung, 1 × 10^5^ BAL cells were collected and stained with a combination of A5-APC, CD45.2-FITC/PerCP Cy5.5, CD11b-PE Cy7, CD11c-APC Cy7, CD14-PerCP Cy5.5, Ly6G-PE, and NP-FITC. All flow cytometry was performed using the BD FACSCanto II flow cytometer and sorting was performed using the FACS ARIA III (BD Biosciences). Data were analysed using FlowJo software 8.8.10 (Tree Star). ApoBD formation index is calculated by number of A5^+^ ApoBDs/number of apoptotic cells (or A5^+^ cells).

### ApoBD isolation

ApoBDs were purified from either UV-irradiated or IAV-infected THP1 monocytes, or primary BAL samples by methods previously described using either a differential centrifugation-based approach, or a FACS-based approach^15,17^. In brief, using the Beckman Coulter Allegra® X-15R Centrifuge, ApoBDs were isolated through differential centrifugation by performing an initial centrifugation step at 300 × *g* for 10 min to remove cells. The supernatant was subsequentially centrifuged at 3000 × *g* for 20 min to pellet ApoBDs, whereas smaller EVs remain in the supernatant.

For THP1 FACS-based ApoBD isolations, UV-irradiated (2–4 h post UV) and IAV-infected (24 h p.i.) THP1 cells were collected, and pelleted at 3000 × *g* for 6 min. Samples were stained with A5 and TO-PRO-3 for 10 min, centrifuged (3000 × *g* for 6 min), and resuspended in sorting buffer containing 1× PBS, 2 mM EDTA, 1× A5 binding buffer, and 10% FSC. Samples were then filtered through a 70 µm cell strainer prior to FACS. Gating strategies were first established by defining ApoBDs as FSC^low^, SSC^low^, TO-PRO-3^low/intermediate^, and A5^+^ and the sorting efficiency was first validated by performing a test sort to confirm purity. After sorting, ApoBDs were centrifuged at 3000 × *g* for 6 min to pellet ApoBDs before subsequent analysis. A similar FACS-based approach was also implemented for the isolation of primary mouse monocyte-derived ApoBDs, where BAL samples were collected, pelleted (3000 × *g* for 6 min), and resuspended in an antibody cocktail where ApoBDs were identified as A5^+^, PO-PRO-1^low/intermediate^, CD45.2^+^, Ly6G^−^, F4/80^−^, CD64^+^ FSC^low^, and SSC^low^. Notably, all ApoBD samples were centrifuged at 3000 × *g* as this is sufficient to pellet ApoBDs; however, free virions (including larger filamentous virions) are likely to remain in the discarded supernatant.

### DNA fragmentation assay

To confirm the induction of apoptosis, DNA fragmentation was performed as per Atkin-Smith et al. Briefly, 1 × 10^6^ untreated or PR8-infected cells were collected (24 h p.i.), resuspended in TES lysis buffer with RNAase, and incubated for 2 h at 37 °C. Lysates were then treated with proteinase K for 16 h at 50 °C and DNA was separated by gel electrophoresis.

### Immunoblotting

Samples were lysed in cytobuster protein extraction reagent (Novagen, Germany) and analysed by SDS–PAGE. Immunoblotting was performed using the following antibodies and dilutions: rabbit anti-pro-caspase 3 (1:2000, Santa Cruz), rabbit antisera anti-PB1 (1:1000, gift from Dr Jonathan Yewdell, NIAID, NIH Bethesda, MD, USA), mouse anti-HA^50^ (1:1000, gift from Dr Jonathan Yewdell), mouse anti-β-actin (1:4000, Sigma-Aldrich,) horseradish peroxidase-conjugated sheep anti-mouse Ig (1:4000, GE Healthcare), and horseradish peroxidase-conjugated donkey anti-rabbit (1:4000, GE Healthcare).

### Time-lapse DIC microscopy

All DIC microscopy analysis was performed using the Zeiss Spinning Disk Confocal microscope (Zeiss, Oberkochen, Germany) with 63× oil-immersion objective and four-well Nunc Lab-Tek II chambered coverglass slides (Thermo Fisher Scientific, Massachusetts, USA). For disassembly analysis of UV-treated THP1 monocytes, cells were added to chambers pre-coated with 1% poly-l-lysine (Sigma-Aldrich) and allowed to adhere before UV irradiation. Time-lapse microscopy was performed by collecting images every 2 min over 4 h at 37 °C, 5% CO_2_. Imaging of PR8-infected THP1 monocytes was performed by collecting images ~24 h p.i. All imaging processing and data analysis were performed using ZEN imaging software (Zeiss).

### Confocal microscopy

PR8-infected THP1 monocytes were first stained with NP-FITC or HA-FITC (~24 h p.i.) and added to four-well Nunc Lab-Tek II chambered coverglass slides. For GFP-PR8 analysis, cells were infected and added directly to chambers for imaging 24 h p.i. For imaging of IAV-infected primary BAL cells, samples were added directly to chambers after BAL wash in Hoechst 33324-containing lavage buffer. Cells were incubated at 37 °C, 5% CO_2_ and images were collected using the Zeiss 780 Confocal Microscope, 63× oil-immersion objective (Zeiss).

### PCR and RT-qPCR

For PCR analysis, HA^+^ apoptotic cells and ApoBDs were isolated by FACS, and RNA extraction was performed using a RNAeasy micro kit (Qiagen, Hilden, Germany) as per the manufactures instructions. Next, cDNA synthesis was performed using iSCRIPT cDNA synthesis kit and PCR was performed on ~1 µg cDNA. For RT-qPCR, A549 cells were collected after co-incubation with ApoBDs and RNA was extracted via trizol-based extraction. As per PCR preparation, cDNA synthesis was performed using iSCRIPT and RT-qPCR was performed using Fast Start SYBR Green master mix (Roche Life Sciences, Penzberg, Germany). Finally, NS2 expression from qPCR results was normalised to the house keeping gene, UBC.

### Transmission electron microscopy preparation and imaging

ApoBDs were isolated by differential centrifugation as previously described^17^ and fixed in 3% glutaraldehyde/PBS for 2 h at RT before embedding in 1.5% low melting point agarose. Samples were washed with 0.1 M cacodylate buffer and incubated in 1% OsO4 (in 0.1 M cacodylate buffer) for 40 min at RT. Subsequently, samples were washed once with 0.1 M cacodylate buffer and once in 80% acetone for an incubation overnight at 4 °C in 2% uranyl acetate UAc/80% acetone. The next day, serial dehydrating and resin infiltration steps were performed as follows: 2 × 10 min with 80% acetone, 2 × 10 min with 90% acetone, 3 × 20 min with 100% acetone, 1 × 90 min with 50% Epon/50% acetone, 1 × 90 min with 75% Epon/25% acetone, and 1 × 90 min with 100% Epon. Epon was replaced with fresh 100% Epon with polymerisation accelerator benzyldimethylamine and embedded at 60 °C for 72 h. Resin blocks were then sectioned using a DiATOME 45° Ultra Diamond Knife on a Leica EM UC7—ultramicrotome, and 50 nm thickness sections were obtained and mounted on EM-copper grids with formvar/carbon coating. Sections were post-stained in 4% UAc in water and Reynolds’ lead citrate for 5 min each, and then processed for imaging using a FEI Talos L120C transmission electron microscope.

### Viral plaquing

MDCK epithelial cells were seeded at 0.7 × 10^6^ cells/well of a six well plate. Cells were washed and infected with 500–400 µL of FACS-isolated ApoBDs or PR8 virus in complete (10%) RPMI for 1 h at 37 °C, 5% CO_2_. After incubation, 3–4 mL of agarose/medium (0.4% agarose, 1× Leibovitz L15 medium (Thermo Fisher Scientific), 2 × 10^−4^% Trypsin Worthington), was gently added and incubated for 3 days at 37 °C, 5% CO_2_. Agarose/medium was removed and plaques were counted after PFA fixation and crystal violet staining.

### ApoBD-A549 co-incubation assays

All in vitro ApoBD-A549 co-incubation assays were performed by incubating 1 × 10^5^ FACS-isolated ApoBDs with 1 × 10^5^ target cells (A549) for 48 h at 37 °C in 5% CO_2_, unless otherwise specified. Target cells were then stained by A5-PE and Live Dead-APC or -Yellow, fixed, permeabilised, and stained with NP for flow cytometry analysis.

### ApoBD-virion binding assys

To determine whether virions could bind to apoptotic samples directly, UV-irradiated THP1 monocytes were first incubated with 10 pfu PR8 for either 60 min (the standard procedure for infection) or 15 min (to minimise viral endocytosis) in acidified media. ApoBDs were then analysed by flow cytometry to confirm HA-FITC expression (indicative of virion binding). For subsequent assays using virion-bound ApoBDs, ApoBDs from the 15 min PR8 incubation were isolated via our FACS-based approach, washed three times (centrifuging at 3000 × *g*, 6 min) and then used in co-incubation assays, as described above.

### Lung titre and cytometric bead array

To measure ApoBD-mediated viral propagation in vivo, B6 mice with treated with either 1000 pfu PR8 or FACS-isolated ApoBDs (0.4–1 × 10^5^) as described above. Mice were sacrificed on day 3 post treatment, and lung tissue was harvested and homogenised. Lung viral titres in the lungs were determined using plaque assays on MDCK cells as previously described^51^. Inflammatory cytokine release were quantified on lung homogenates using a cytometric bead array kit (BD Biosciences) according to the manufacturer’s instructions and as previously described^51^.

### In vivo ICS and inflammatory cell analysis

FACS-isolated ApoBDs (1 × 10^5^) were administered i.n. (as specified above), and mice were sacrificed at days 7–8 and the BAL, lung tissue, and spleen were harvested. For ex vivo ICS, 100,000–300,000 lung and spleen cells were collected, and stimulated with 6 × 10^−6^ M peptide (HA_211–225_, NP_311–325_, PA_224–233_, NP_366–374_, PB1_703–711_, and PB1F2_62–70_) for 1 h and treated with 10 µg/mL Brefeldin A for 4 h at 37 °C in 5% CO_2_. Samples were then stained with CD3-APC, CD4-APC Cy7, CD8-BV410, Live Dead-Aqua, fixed (1% PFA), permeabilised (0.2% saponin), blocked (0.2% saponin and 1% BSA), and ICS was performed with TNF-α-FITC and IFN-γ-PE. For lung inflammatory cell analysis, 100,000 BAL cells were collected and stained with CD45.2-FITC, Ly6G-PE, CD11c-APC Cy7, CD11b-PE Cy7, and DAPI.

### Pharmacological inhibition of apoptotic cell disassembly

For in vitro analysis, Halo was dissolved in DMSO to 20 mM stocks. Titrations were performed by incubating 1.5 × 10^5^ THP1 monocytes with varying concentrations of Halo in 1% BSA, RPMI for 4 h. Flow cytometry was then performed as per Jiang et al. to determine the levels of viable, apoptotic, and necrotic cells^49^. To determine whether impairing apoptotic cell disassembly through Halo treatment could alter viral propagation to neighbouring cells, 1 × 10^5^ THP1 monocytes were infected with PR8, washed three times in PBS, and incubated with 3 × 10^5^ A549 epithelial cells for 48 h. Staining and flow cytometry was performed as above. For in vivo assays, Halo was dissolved in 100% acetic acid and diluted in PBS. Mice were infected with 1000 pfu PR8 (as detailed above) and treated with either 0.5% acetic acid or 3 mg/kg Halo i.n., 2 days p.i. For apoptosis analysis, the BAL wash was collected on day 3 p.i. and stained as per ‘Flow cytometry and FACS’.

### Histology

For histological analysis, mice were scarified on day 6 p.i. (day 4 post Halo administration) or ethical endpoint, and lungs were inflated and fixed using 4% PFA for 24 h. Lungs were then resuspended in 70% ethanol for at least 1 week before tissue processing (TP1020, Leica Biosystems) for ~12 h as follows: 70% ethanol, 1 h; 100% ethanol, 1 h; 100% ethanol, 1 h; 100% ethanol 1.5 h; 100% ethanol, 1.5 h; xylene, 1 h; xylene 1 h; xylene, 1 h; xylene, 1.5 h; paraffin wax (60 °C), 1 h; paraffin wax (60 °C), 1 h; and paraffin wax (60 °C), 1 h. Samples were embedded in paraffin wax and 5-µm thick whole lung sections were obtained using the microtome, and stained with haemotoxylin and eosin. Tiled photomicrographs of whole lung sections were captured using x10 Nikon Eclipse microscope and haemorrhaging was scored blind (Supplementary Fig. 13).

### Statistics and reproducibility

Unless otherwise specified, data are presented as mean ± the standard error of the mean (SEM). To determine statistical significance between two groups (control versus a specific treatment), unpaired Student’s two-tailed *t*-test was applied. A *P* value of <0.05 was considered statistically significant. Sample sizes and number of replicates is specified within the figure legends.

### Reporting summary

Further information on research design is available in the Nature Research Reporting Summary linked to this article.

## Supplementary information

Supplementary Information

Description of Additional Supplementary Files

Supplementary Data 1

Peer Review File

Reporting Summary

## Data Availability

Presented raw data are available in Supplementary Data 1. Additional manuscript data is available from the authors upon request to g.atkin-smith@latrobe.edu.au or i.poon@latrobe.edu.au.
