# Supplementary material for: Monocyte apoptotic bodies are vehicles for influenza A virus propagation
Source: Commun Biol. 2020 May 8;3:223. doi: 10.1038/s42003-020-0955-8 (PMC7210108; doi:10.1038/s42003-020-0955-8)
Supplement: Supplementary file 1 — Supplementary Information [file 42003_2020_955_MOESM1_ESM.pdf]

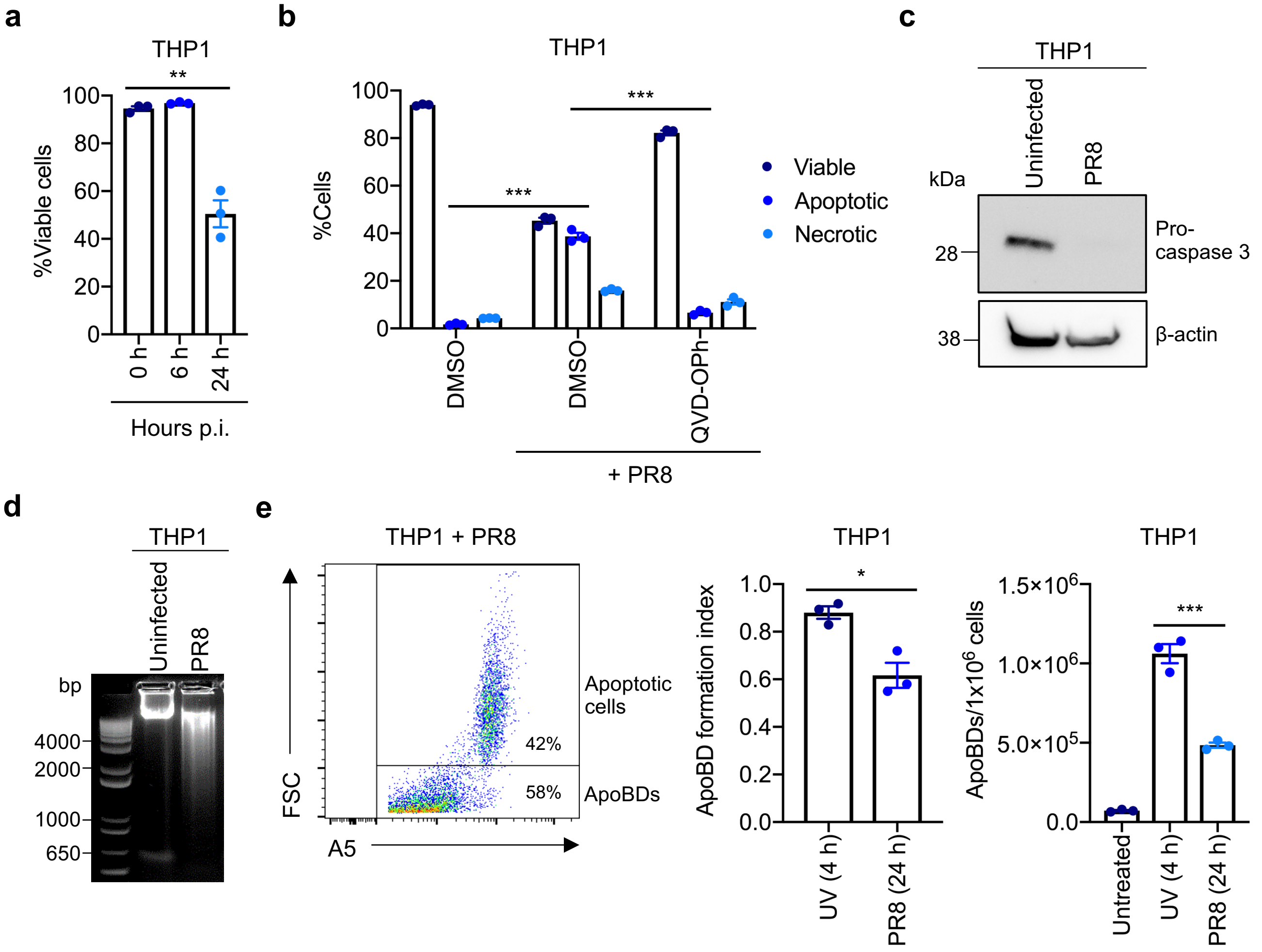

Supplementary Figure 1. IAV induces monocyte apoptosis. (a) THP1 monocytes were infected with the IAV strain PR8 at a MOI of 10 for 6 or 24 h and viable cells were determined by flow cytometry. Data represents three independent repeats. **(b)** PR8-infected THP1 monocytes were treated with or without 50 µM Q-VD-OPh for 24 h and viable, apoptotic and necrotic cells were quantified by flow cytometry (24 h p.i.). THP1 monocytes were infected with PR8 and subjected to; **(c)** immunoblot analysis to determine pro-caspase 3 cleavage or **(d)**, DNA fragmentation assay (representative of n=2 independent repeats). **(e)** THP1 monocytes were subjected to UV irradiation (4 h), infected with PR8 (24 h) or untreated and ApoBD formation was assessed by flow cytometry to determine the ApoBD formation index and the absolute number of ApoBDs/1x10^6 cells. Unless specified otherwise, data is representative of 3 independent experiments where error bars represent SEM of n=3 biological repeats,* = P<0.01 ** = P<0.01, *** = P<0.001, unpaired Student's two tailed t-test.

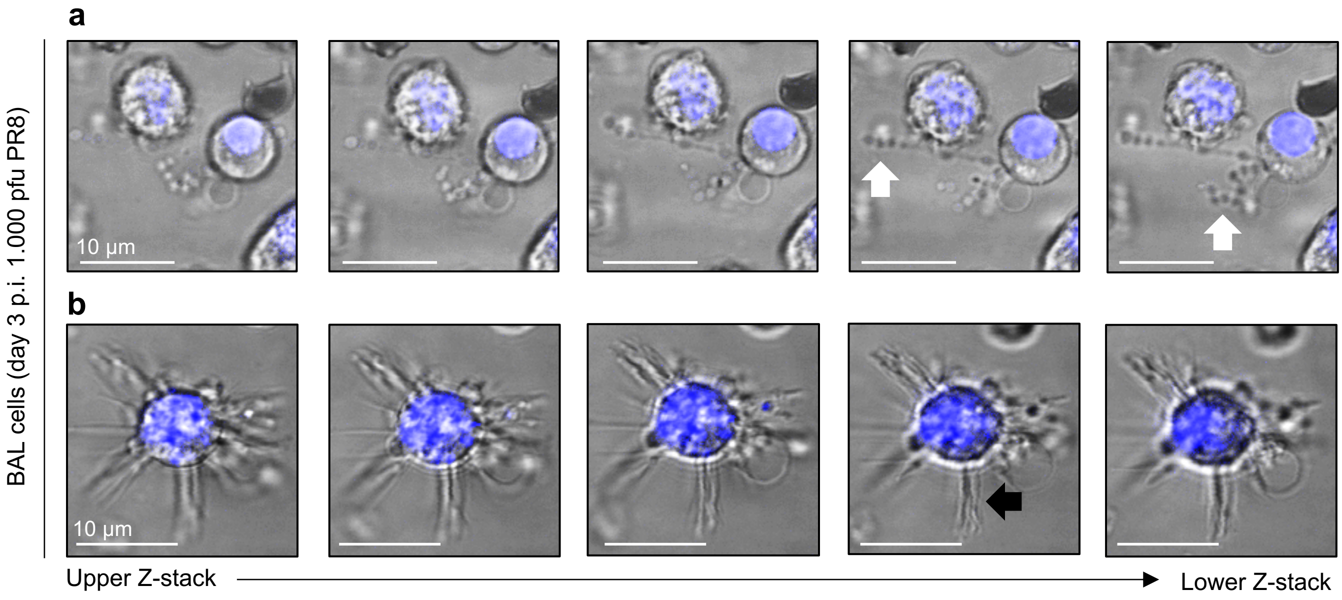

Supplementary Figure 2. Apoptotic BAL cells from PR8-infected mice generate apoptopodia. B6 mice were infected with 1,000 pfu PR8 and BAL cells were collected, stained with DAPI and imaged by confocal microscopy, 3 days p.i. (**a**, **b**) Representative z-stack images of apoptotic BAL cells. White arrows refer to beaded apoptopodia, black arrows refer to non-beaded apoptopodia.

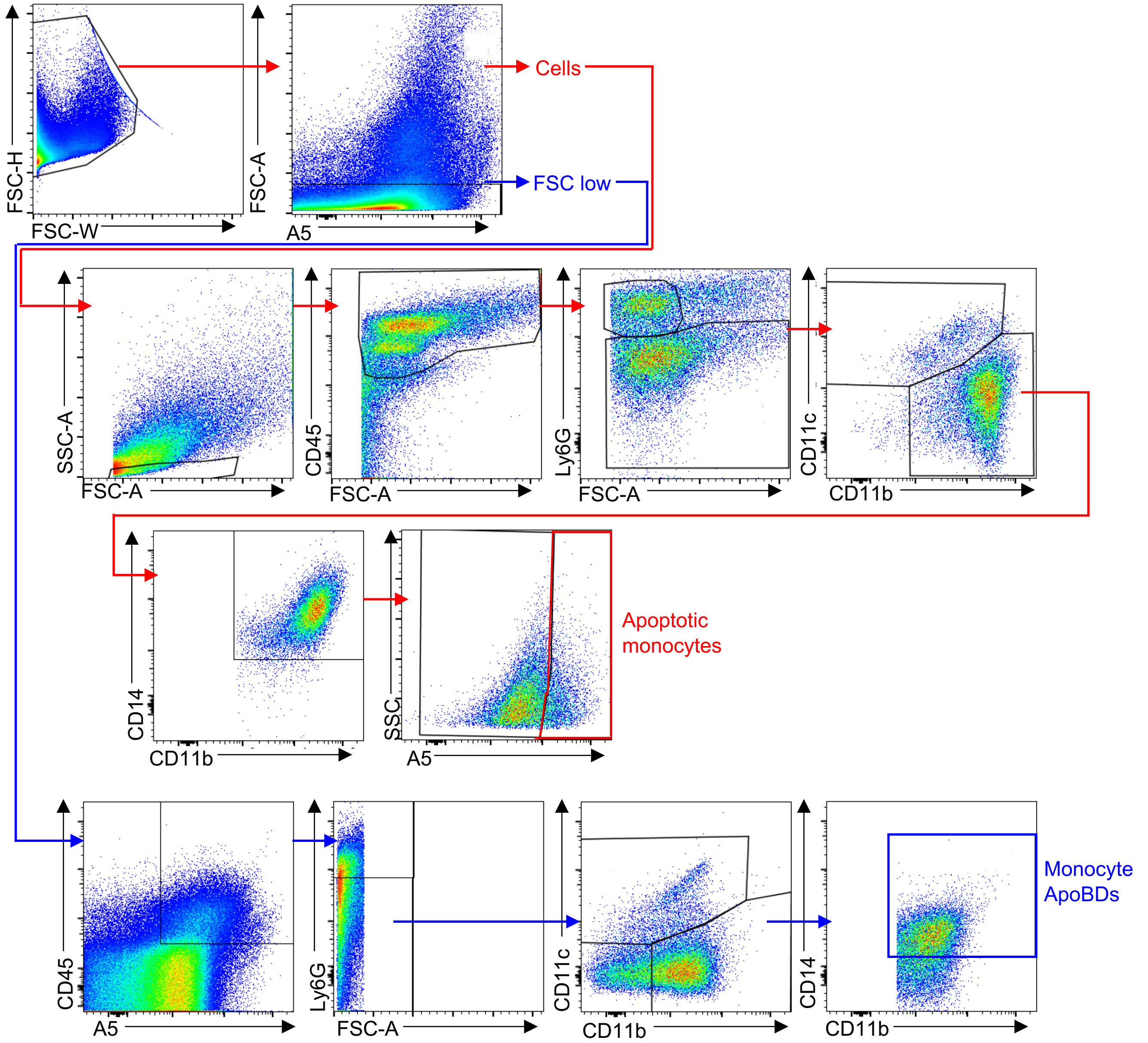

Supplementary Figure 3. Gating strategy used to identify and isolate primary mouse monocyte ApoBDs from BAL. Red arrows indicate work flow to identify cells including apoptotic monocytes. Blue arrows indicate work flow to identify ApoBDs such as monocyte-derived ApoBDs. Representative FACS plots from an IAV infected mouse.

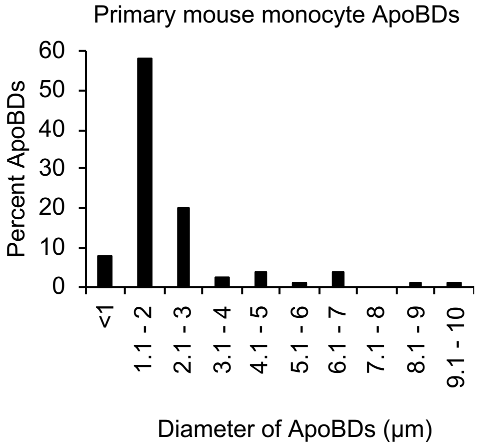

Supplementary Figure 4. Determining the size of mouse monocyte ApoBDs. Primary mouse monocyte ApoBDs were isolated via a FACS-based approach from 1,000 pfu PR8 treated C57/B6 mice, day 3 p.i. The diameter of ApoBDs were quantified via DIC confocal microscopy, (N = 79).

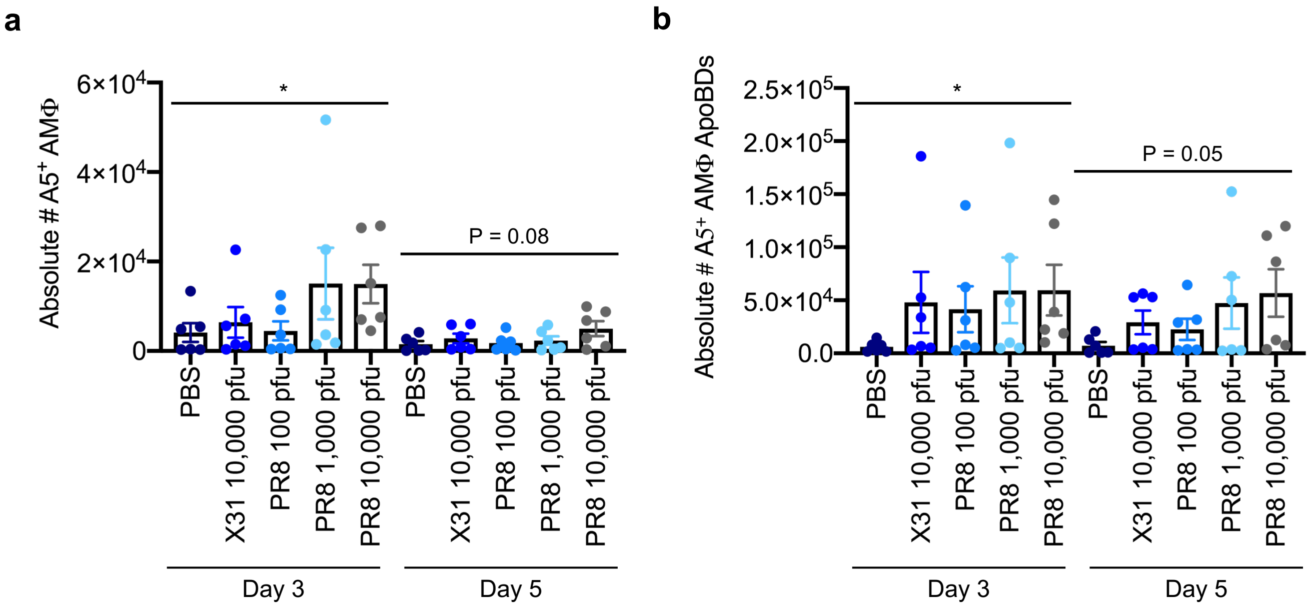

Supplementary Figure 5. PR8 infection induces AMΦ apoptotic cell disassembly. The relative number of A5+ AMΦs and AMΦ-derived ApoBDs were quantify by flow cytometry (**a**, **b**), day 3 and day 5 post PBS or IAV administration. Error bars represent SEM of n=6 mice, * = P <0.05, Student's unpaired two-tailed t-test.

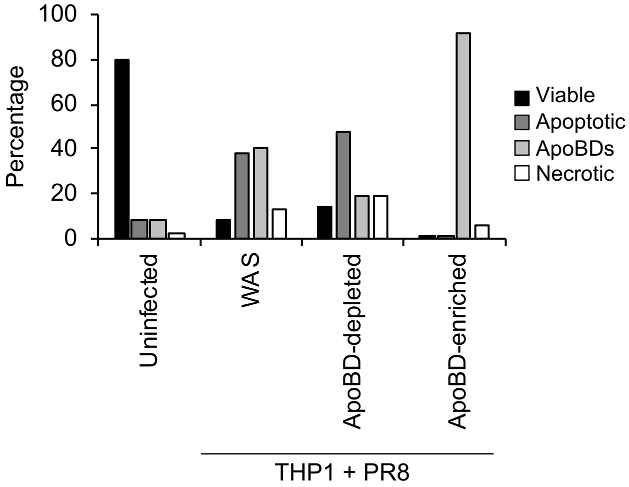

Supplementary Figure 6. THP1 ApoBDs can be isolated by differential centrifugation. Flow cytometry analysis of THP1 monocyte ApoBDs isolated by a differential centrifugation-approach was performed to validate the percentage of viable, apoptotic, necrotic cells and ApoBDs. Data is representative of 2 independent repeats.

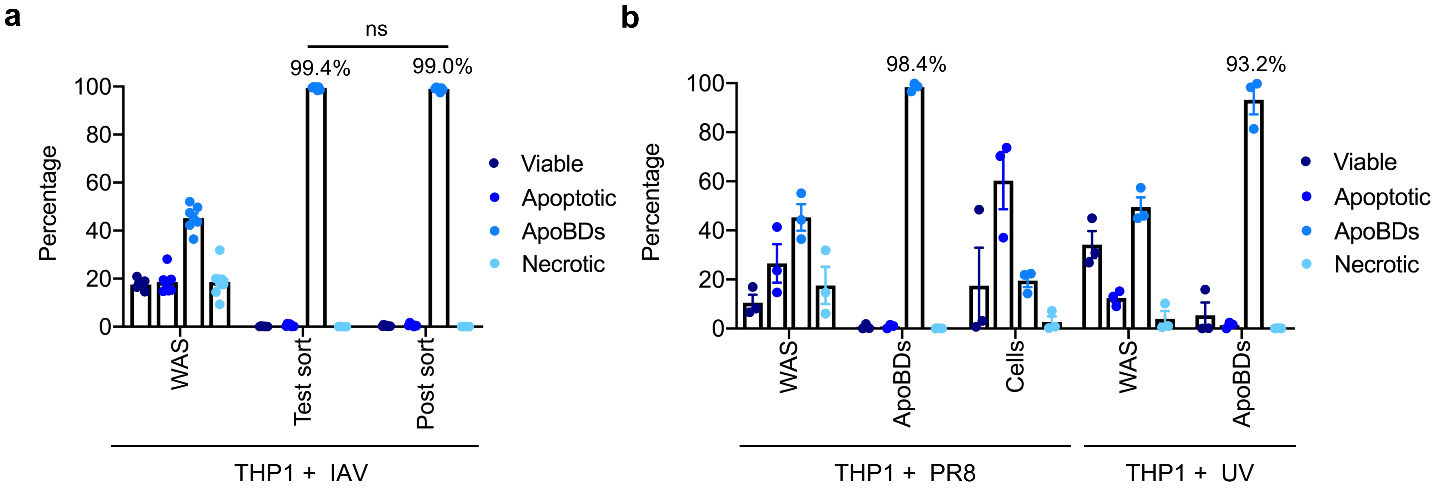

Supplementary Figure 7. Validation of FACS-based ApoBD isolation approach. a The purity of FACS-isolated ApoBDs was confirmed between test and post-sort samples. **b** The levels of viable, apoptotic and necrotic cells and ApoBDs was determined to confirm the sorting efficiency of the FACS-based isolation approach used to isolate ApoBDs for MDCK-viral plaquing. Error bars represent SEM of n=3 independent repeats, ns = P>0.05, unpaired Student's two tailed t-test.

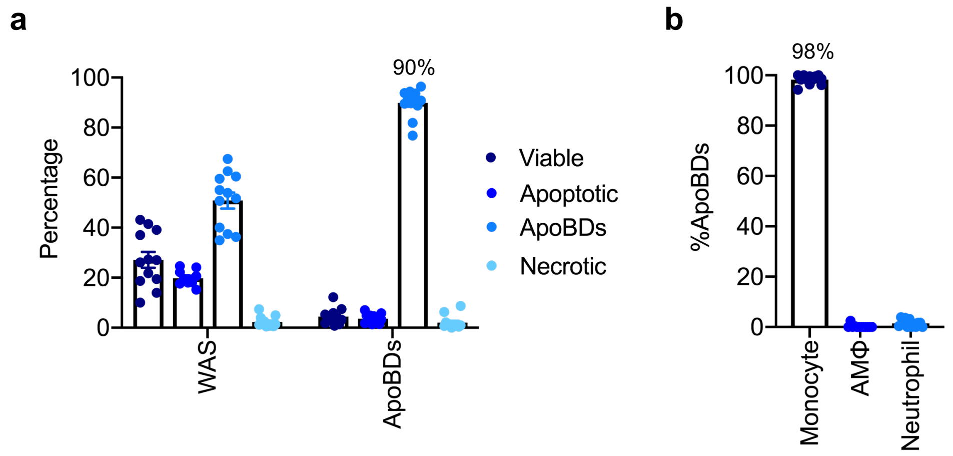

Supplementary Figure 8. Primary monocyte ApoBDs can be isolated by FACS. Primary mouse monocyte ApoBDs were isolated by a FACS-based approach from the BAL of PR8 infected B6 mice (BAL of 1,000 pfu, day 3 p.i.). Flow cytometry was used to determine: (**a**), the percentage of viable, apoptotic and necrotic cells and ApoBDs and (**b**), the cell origins of ApoBDs. Error bars represent SEM of n=12 mice.

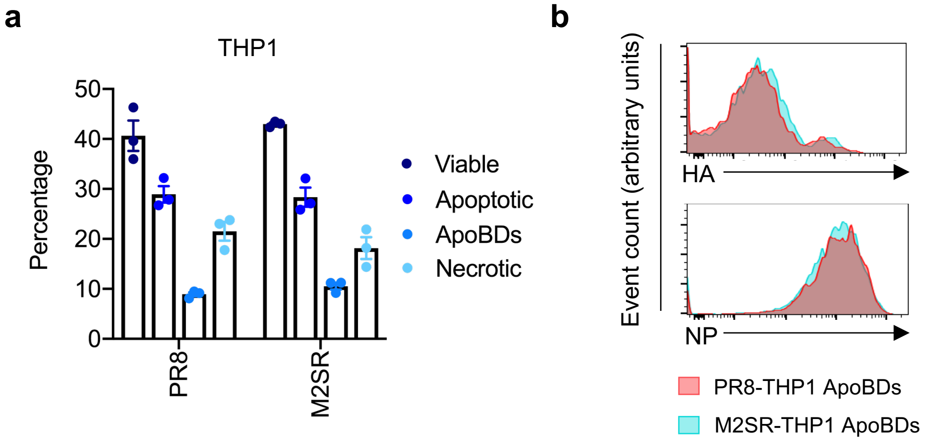

Supplementary Figure 9. M2SR virus induces comparable levels of cell death and expression of viral proteins. a THP1 monocytes were infected with either PR8 or M2SR at a MOI of 10 and flow cytometry was used to determine the levels of viable, apoptotic and necrotic cells and ApoBDs, 24 h p.i. **b** THP1 monocytes were infected with PR8 or M2SR at a MOI of 10 and flow cytometry was used to determine the expression of HA and NP on ApoBDs, 24 h p.i. Data is representative of 3 independent experiments, error bars represent SEM of n=3 biological repeats.

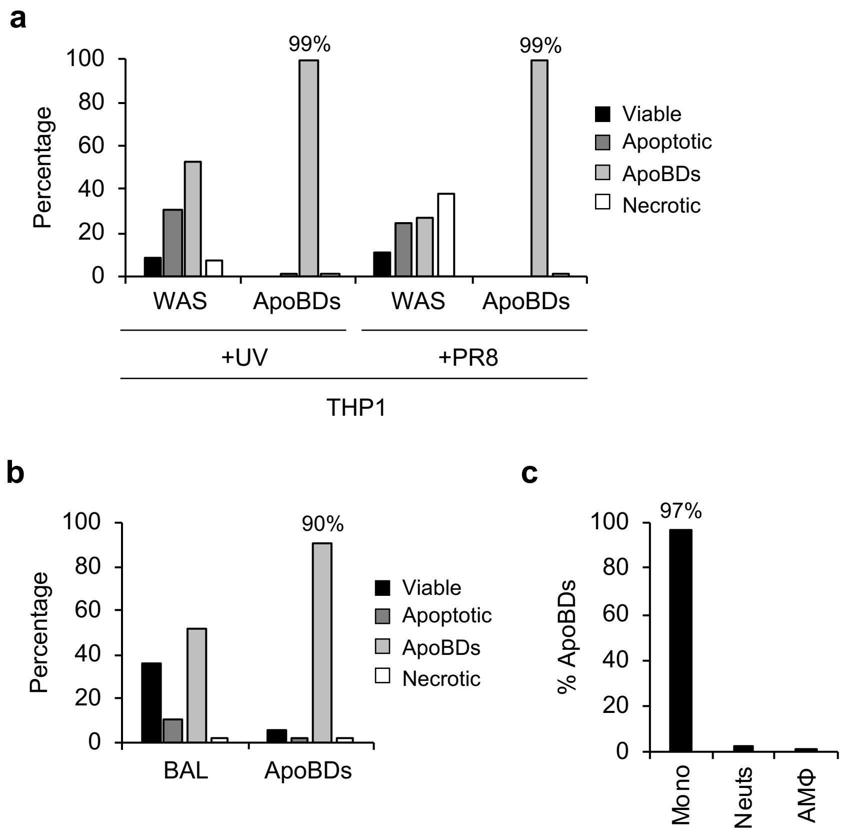

Supplementary Figure 10. FACS-based isolation of ApoBDs for *in vivo* viral propagation analysis. a The sorting efficiency of the FACS-based ApoBD isolation approach was validated for UV irradiated or PR8 infected THP1 monocytes. Primary mouse monocyte ApoBDs were isolated by a FACS-based approach from the BAL of PR8 infected B6 mice (1,000 pfu, day 3 p.i.). Flow cytometry was performed to determine the the percentage of viable, apoptotic and necrotic cells and ApoBDs (**b**), and the cell origins of ApoBDs (**c**).

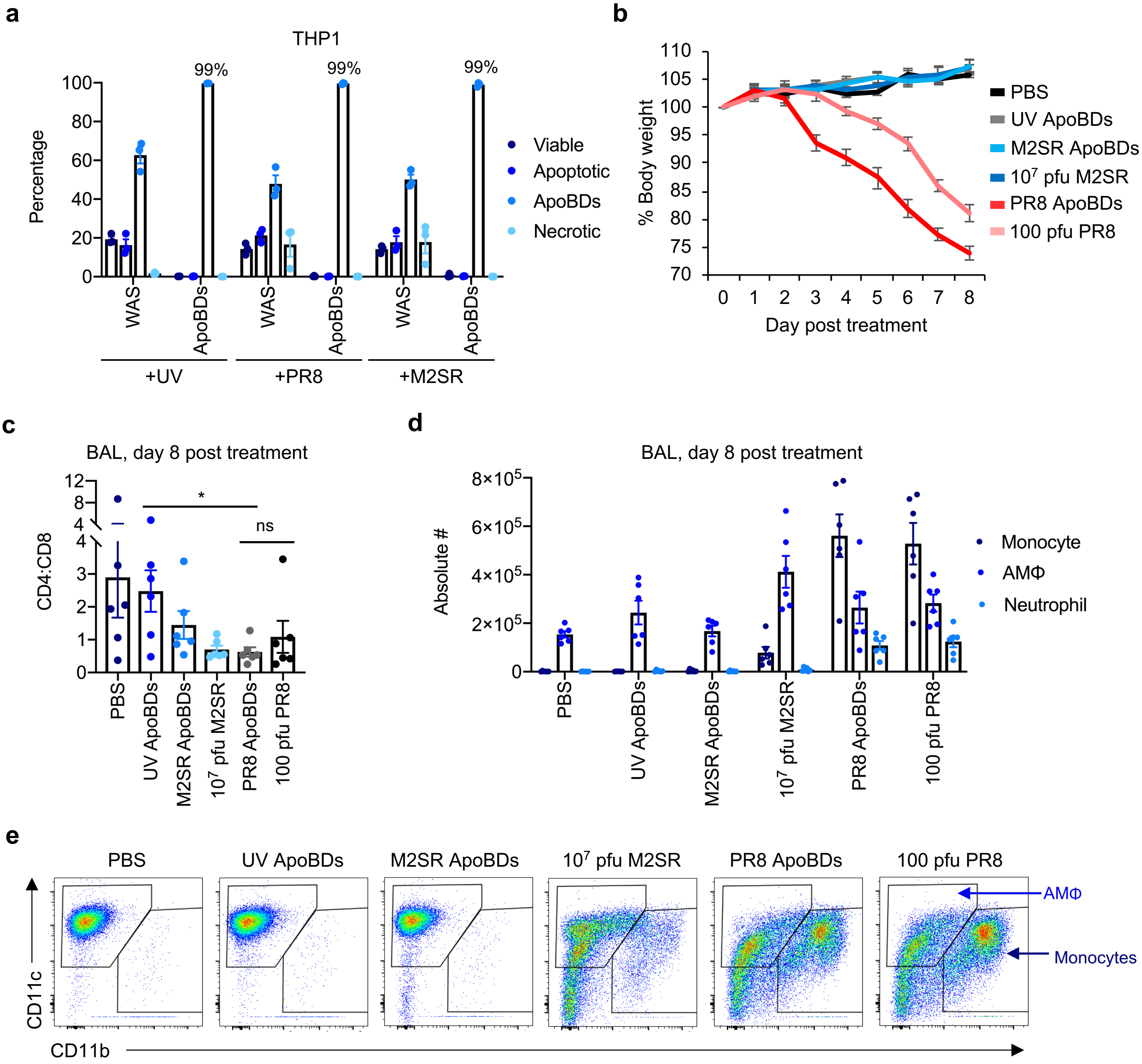

Supplementary Figure 11. THP1-PR8 ApoBDs mediate viral infection *in vivo*. **a** The sorting efficiency of a FACS-based ApoBD isolation approach was validated for UV irradiated, PR8 infected or M2SR infected THP1 monocytes. Error bars represent SEM of n=3 independent repeats. **b** B6 mice were administered with either PBS, THP1-UV ApoBDs, THP1-M2SR ApoBDs, 10^7 pfu M2SR, THP1-PR8 ApoBDs or 100 pfu PR8 and weighed daily for 8 days. The BAL was harvested and the CD4:CD8 T cell ratio (**c**) and inflammatory cell infiltration (**d**, **e**) was determined by flow cytometry. **b-d** Error bars represent SEM of n=6 mice, * = P<0.01, unpaired Student's two tailed t-test.

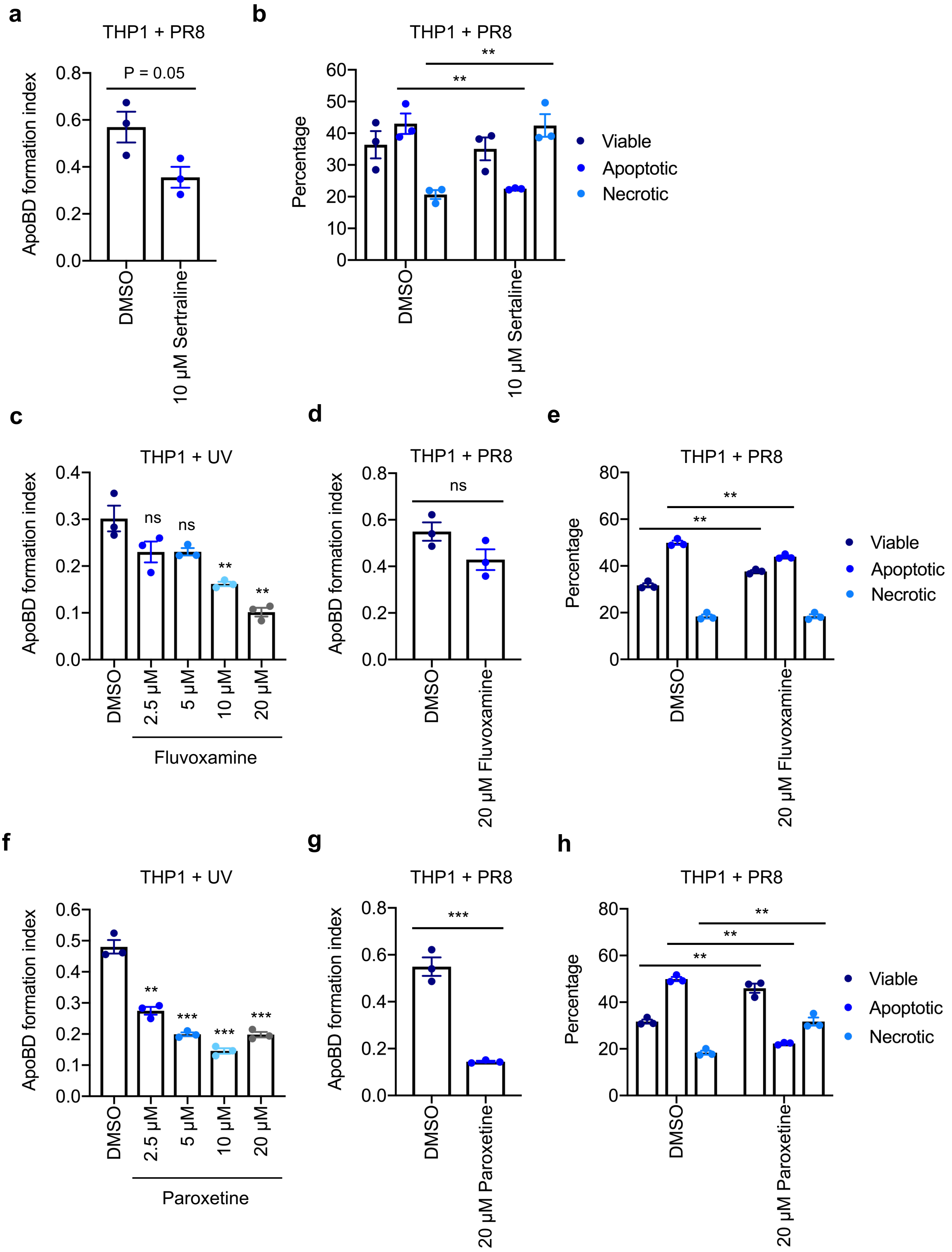

Supplementary Figure 12. Testing the ability of antidepressants to inhibit THP1 apoptotic cell disassembly. PR8-infected THP1 monocytes were treated with 20 μM Sertraline and flow cytometry was used to determine the ApoBD formation index (**a**), and the levels of viable, apoptotic and necrotic cells (**b**). UV-irradiated THP1 monocytes were treated with increasing concentrations of either Fluvoxamine or Paroxetine and the ApoBD formation index was determined by flow cytometry (**c**, **f**). PR8-infected THP1 monocytes were treated with 20 μM Fluvoxamine or Paroxetine and flow cytometry was used to determine the ApoBD formation index (**d**, **g**) and the levels of viable, apoptotic and necrotic cells (**e**, **h**). Data is representative of 3 independent repeats where error bars represent SEM of n=3 biological repeats,* = *P*<0.01 ** = *P*<0.01, *** = P<0.001, unpaired Student's two tailed *t*-test.

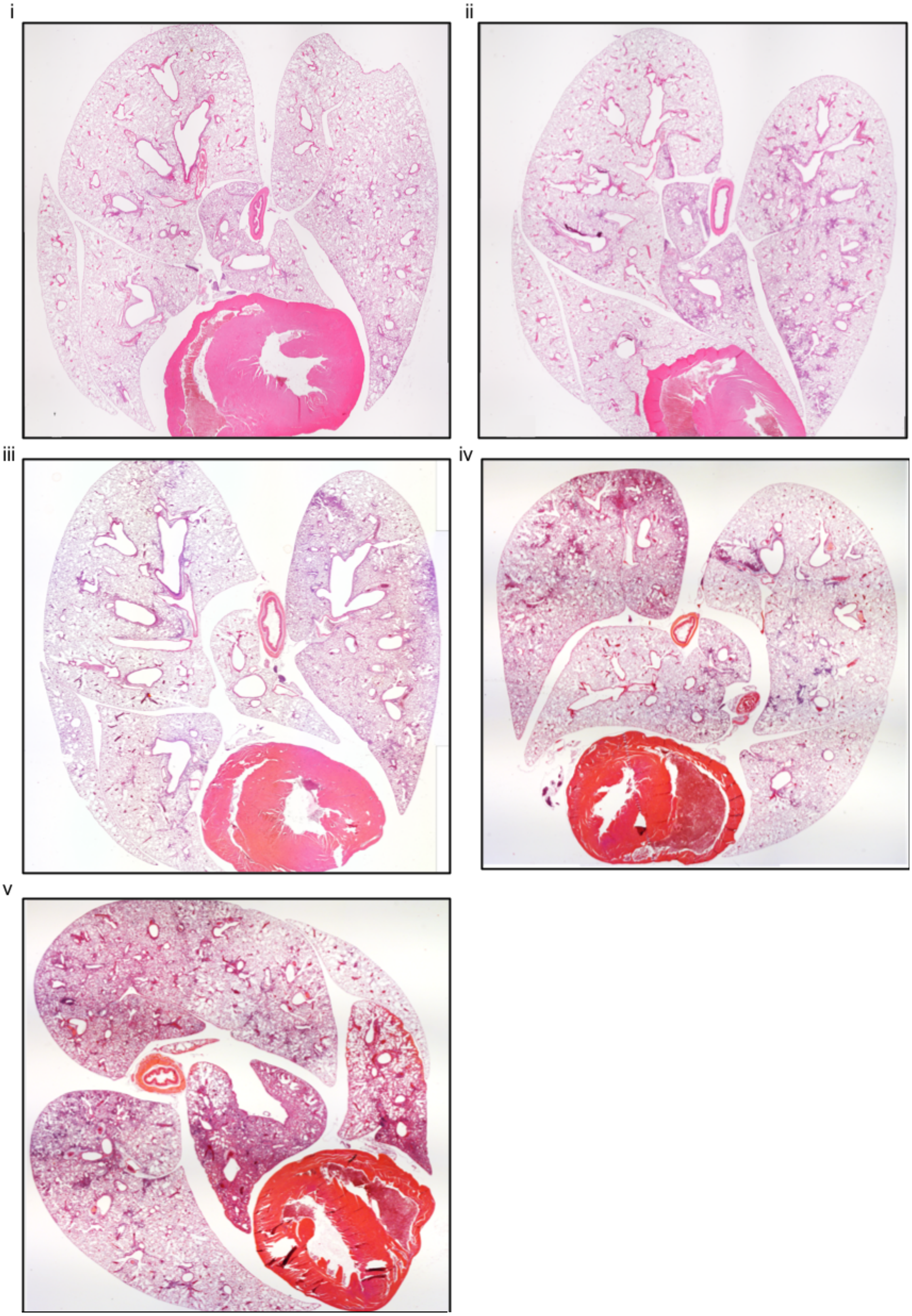

Supplementary Figure 13. Haemorrhaging scoring reference. Histological analysis was performed on paraffin-embedded, H&E stained lung sections to determine the degree of tissue damage and scored blind through a semi-quantitative approach as either i: no haemorrhaging, ii: mild haemorrhaging in one lobe, iii: mild haemorrhaging in multiple lobes, iv: severe haemorrhaging in one lobe or, v: severe haemorrhaging in multiple lobes.
