# Supplementary material for: Monocyte apoptotic bodies are vehicles for influenza A virus propagation
Source: Commun Biol. 2020 May 8;3:223. doi: 10.1038/s42003-020-0955-8 (PMC7210108; doi:10.1038/s42003-020-0955-8)
Supplement: Supplementary file 2 — Description of Additional Supplementary Files [file 42003_2020_955_MOESM2_ESM.pdf]

Description of Additional Supplementary Files

File Name: **Supplementary Data 1**
Description: Raw data for all presented data in the main Figures 1-6 and Supplementary Figures 1-12.
