# Supplementary material for: Monocyte apoptotic bodies are vehicles for influenza A virus propagation
Source: Commun Biol. 2020 May 8;3:223. doi: 10.1038/s42003-020-0955-8 (PMC7210108; doi:10.1038/s42003-020-0955-8)
Supplement: Supplementary file 5 — Reporting Summary [file 42003_2020_955_MOESM5_ESM.pdf]

nature research

Corresponding author(s): Ivan KH Poon, Weisan Chen, Mubing Duan

Last updated by author(s): Apr 14, 2020

Reporting Summary

Nature Research wishes to improve the reproducibility of the work that we publish. This form provides structure for consistency and transparency in reporting. For further information on Nature Research policies, see Authors & Referees and the Editorial Policy Checklist.

Statistics

For all statistical analyses, confirm that the following items are present in the figure legend, table legend, main text, or Methods section.

n/a	Confirmed	
☐	☒	The exact sample size (n) for each experimental group/condition, given as a discrete number and unit of measurement
☐	☒	A statement on whether measurements were taken from distinct samples or whether the same sample was measured repeatedly
☐	☒	The statistical test(s) used AND whether they are one- or two-sided<br>*Only common tests should be described solely by name; describe more complex techniques in the Methods section.*
☐	☒	A description of all covariates tested
☒	☐	A description of any assumptions or corrections, such as tests of normality and adjustment for multiple comparisons
☐	☒	A full description of the statistical parameters including central tendency (e.g. means) or other basic estimates (e.g. regression coefficient) AND variation (e.g. standard deviation) or associated estimates of uncertainty (e.g. confidence intervals)
☐	☒	For null hypothesis testing, the test statistic (e.g. F, t, r) with confidence intervals, effect sizes, degrees of freedom and P value noted<br>*Give P values as exact values whenever suitable.*
☒	☐	For Bayesian analysis, information on the choice of priors and Markov chain Monte Carlo settings
☒	☐	For hierarchical and complex designs, identification of the appropriate level for tests and full reporting of outcomes
☒	☐	Estimates of effect sizes (e.g. Cohen's d, Pearson's r), indicating how they were calculated

Our web collection on statistics for biologists contains articles on many of the points above.

Software and code

Policy information about availability of computer code

Data collection	Zeiss Zen 2 Blue Edition, Zeiss Zen 2011 SP Black Edition, BD FACS Canto Software
Data analysis	GraphPad Prism Version 8.1.0 (221), FlowJo Version 10.5.3

For manuscripts utilizing custom algorithms or software that are central to the research but not yet described in published literature, software must be made available to editors/reviewers. We strongly encourage code deposition in a community repository (e.g. GitHub). See the Nature Research guidelines for submitting code & software for further information.

Data

Policy information about availability of data

All manuscripts must include a data availability statement. This statement should provide the following information, where applicable:

- Accession codes, unique identifiers, or web links for publicly available datasets
- A list of figures that have associated raw data
- A description of any restrictions on data availability

Presented raw data is available in the Supplementary Data File. Additional manuscript data is available from the authors upon request to g.atkin-smith@latrobe.edu.au or, i.poon@latrobe.edu.au.

Field-specific reporting

Please select the one below that is the best fit for your research. If you are not sure, read the appropriate sections before making your selection.

☒ Life sciences ☐ Behavioural & social sciences ☐ Ecological, evolutionary & environmental sciences

Life sciences study design

All studies must disclose on these points even when the disclosure is negative.

Sample size	Sample size of at least n=3 was utilised as previously described
Data exclusions	No data was excluded
Replication	Unless otherwise specified, data is representative of at least three independent repeats
Randomization	NA
Blinding	Blinding occurred exclusively for scoring of histological data

Reporting for specific materials, systems and methods

We require information from authors about some types of materials, experimental systems and methods used in many studies. Here, indicate whether each material, system or method listed is relevant to your study. If you are not sure if a list item applies to your research, read the appropriate section before selecting a response.

Materials & experimental systems

n/a	Involved in the study	
☐	☒	Antibodies
☐	☒	Eukaryotic cell lines
☐	☐	Palaeontology
☐	☒	Animals and other organisms
☐	☐	Human research participants
☐	☐	Clinical data

Methods

n/a	Involved in the study	
☐	☐	ChIP-seq
☐	☒	Flow cytometry
☐	☐	MRI-based neuroimaging

Antibodies

Antibodies used

Immunoblotting was performed using the following antibodies and dilutions: rabbit anti-pro-caspase 3 (1:2000, Santa Cruz), rabbit antisera anti-PB1 (1:1000, gift from Dr Jonathan Yewdell, NIAID, NIH Bethesda, MD, USA), mouse anti-HA50 (1:1000, gift from Dr Jonathan Yewdell), mouse anti-β-actin (1:4000, Sigma-Aldrich,) horseradish peroxidase-conjugated sheep anti-mouse Ig (1:4000, GE Healthcare) and horseradish peroxidase-conjugated donkey anti-rabbit (1:4000, GE Healthcare).

The following regents were purchased from BD Biosciences (Auckland, New Zealand): A5-FITC, -PE, -APC, mouse CD4-APC Cy7, -PE Cy7, anti-mouse CD3-APC, anti-mouse CD45.2 PerCP Cy5.5, anti-mouse Ly6G-PE, TNFα-FITC. Anti-mouse CD8a-e450, anti-mouse CD11b-PE Cy7, NP-FITC, were purchased from Thermofisher (Scoresby, Australia). Anti-mouse CD14-PerCP Cy5.5, anti-mouse CD45.2-FITC, anti-mouse CD8-PE and anti-mouse CD11c-APC Cy7 were purchased form Biolegend (San Diego, USA). HA-FITC was purchase from Santa Cruz Biotehnology (Texas, USA) and NP-FITC was purchased from Invitrogen (Carlsbad, USA). Anti-IFN-y was purchased from Tonbo Biosciences (Ferris Square, USA)

Eukaryotic cell lines

Policy information about cell lines

Cell line source(s)

THP1 monocytes and A549 epithelial cells were cultured in complete (10%) RPMI consisting of RPMI 1640 medium, 50 IU/mL penicillin and 50 µg/mL streptomycin mixture, and 10% (vol/vol) FSC. THP1 monocytes were also cultured in the presence of 0.2% (vol/vol) MycoZap reagent. Primary human monocytes were isolated from whole blood from healthy donors (Australian Red Cross Blood Service, agreement number: 14-11 VIC-03, La Trobe University Human Ethics: FHEC09/R16) through Filcol isolation and CD14 MicroBeads, in accordance to the manufactures instructions (Miltenyi Biotec, Bergisch Gladbach, Germany). All cell lines were incubated at 37°C in 5% CO_2.

Animals and other organisms

Policy information about studies involving animals; ARRIVE guidelines recommended for reporting animal research

Laboratory animals	Female C57BL/6 mice were purchased from the Walter Eliza Hall Institute of Medical Research (WEHI) (Melbourne, Australia). All mice were housed in specific pathogen-free (SPF) isolators and infected with IAV at 6-8 weeks of age under ethics approval of AEC15-84.
Ethics oversight	All experiments were approved by the La Trobe University Animal Ethics Committee in accordance with the National Health and Medical Research Council Australia code of practice for the care and use of animals for scientific purposes.

Flow Cytometry

Plots

Confirm that:

☒ The axis labels state the marker and fluorochrome used (e.g. CD4-FITC).

☒ The axis scales are clearly visible. Include numbers along axes only for bottom left plot of group (a 'group' is an analysis of identical markers).

☒ All plots are contour plots with outliers or pseudocolor plots.

☒ A numerical value for number of cells or percentage (with statistics) is provided.

Methodology

Sample preparation	Samples were prepared as previously described.
Instrument	BD FACS Canto II, BD FACS ARIA III
Software	FlowJo
Cell population abundance	Please refer to supplementary figure 7
Gating strategy	Gating was performed as per previously described or as per supp figure 3

☒ Tick this box to confirm that a figure exemplifying the gating strategy is provided in the Supplementary Information.
