# Supplementary material for: Monocyte apoptotic bodies are vehicles for influenza A virus propagation
Source: Commun Biol. 2020 May 8;3:223. doi: 10.1038/s42003-020-0955-8 (PMC7210108; doi:10.1038/s42003-020-0955-8)
Supplement: Supplementary file 4 — Peer Review File [file 42003_2020_955_MOESM4_ESM.pdf]

Editorial Note: *This manuscript has been previously reviewed at another Nature Research journal. This document only contains reviewer comments and rebuttal letters for versions considered at Communications Biology*

REVIEWERS' COMMENTS:

Reviewer #1 (Remarks to the Author):

The authors have addressed all of my previous concerns.

Reviewer #2 (Remarks to the Author):

The authors have satisfactorily addressed my previous concerns.
